# Feasibility of a Personal Health Technology-Based Psychological Intervention for Men with Stress and Mood Problems: Randomized Controlled Pilot Trial

**DOI:** 10.2196/resprot.2389

**Published:** 2013-01-09

**Authors:** Päivi Lappalainen, Kirsikka Kaipainen, Raimo Lappalainen, Henna Hoffrén, Tero Myllymäki, Marja-Liisa Kinnunen, Elina Mattila, Antti P Happonen, Heikki Rusko, Ilkka Korhonen

**Affiliations:** ^1^Department of PsychologyUniversity of JyväskyläJyväskylän yliopistoFinland; ^2^VTT Technical Research Centre of FinlandTampereFinland; ^3^Department of Biology of Physical ActivityUniversity of JyväskyläJyväskyläFinland; ^4^Department of Biology of Physical Activity & Department of Health SciencesUniversity of JyväskyläJyväskyläFinland; ^5^Tampere University of Technology, Department of Biomedical Engineering & VTT Technical Research Centre of FinlandTampereFinland

**Keywords:** stress, technology-supported mini-intervention, personal health technologies, cognitive behaviour therapy, acceptance and commitment therapy, mhealth, mobile health, smartphone, Internet

## Abstract

**Background:**

Work-related stress is a significant problem for both people and organizations. It may lead to mental illnesses such as anxiety and depression, resulting in increased work absences and disabilities. Scalable interventions to prevent and manage harmful stress can be delivered with the help of technology tools to support self-observations and skills training.

**Objective:**

The aim of this study was to assess the feasibility of the P4Well intervention in treatment of stress-related psychological problems. P4Well is a novel intervention which combines modern psychotherapy (the cognitive behavioral therapy and the acceptance and commitment therapy) with personal health technologies to deliver the intervention via multiple channels, includinggroup meetings, Internet/Web portal, mobile phone applications, and personal monitoring devices.

**Methods:**

This pilot study design was a small-scale randomized controlled trial that compared the P4Well intervention with a waiting list control group. In addition to personal health technologies for self-assessment, the intervention consisted of 3 psychologist-assisted group meetings. Self-assessed psychological measures through questionnaires were collected offline pre- and post-intervention, and 6 months after the intervention for the intervention group. Acceptance and usage of technology tools were measured with user experience questionnaires and usage logs.

**Results:**

A total of 24 subjects were randomized: 11 participants were followed up in the intervention group (1 was lost to follow-up) and 12 participants did not receive any intervention (control group). Depressive and psychological symptoms decreased and self-rated health and working ability increased. All participants reported they had benefited from the intervention. All technology tools had active users and 10/11 participants used at least 1 tool actively. Physiological measurements with personal feedback were considered the most useful intervention component.

**Conclusions:**

Our results confirm the feasibility of the intervention and suggest that it had positive effects on psychological symptoms, self-rated health, and self-rated working ability. The intervention seemed to have a positive impact on certain aspects of burnout and job strain, such as cynicism and over-commitment. Future studies need to investigate the effectiveness, benefits, and possible problems of psychological interventions which incorporate new technologies.

**Trial Registration:**

The Finnish Funding Agency for Technology and Innovation (TEKES), Project number 40011/08

## Introduction

Work-related stress is one of the biggest health challenges that the world faces at this moment. According to the 2009 European Risk Observatory Report, stress is the second most frequently reported work-related health problem and affects 22% of working Europeans [1]. Long-term exposure to work-related stress has been linked to an increased risk of psychological problems, such as depression, anxiety, emotional exhaustion, and may lead to long-term absenteeism, work disability, and early retirement [2].

Several studies have investigated work-related mental health [3,4]. Psychological interventions based on cognitive behavioral therapies (CBT) have a proven effectiveness for a range of common mental health disorders [5-7]. CBT is also an effective intervention for occupational stress [8-11]. Besides traditional CBT methods, research suggests that stress management interventions based on the acceptance and commitment therapy (ACT) have a positive impact on employees’ psychological health, well-being, and stress management skills [12-19]. Research implicates that psychological acceptance promoted by ACT is associated with not only mental health variables but also with a performance-related variable.

Lifestyle-related chronic conditions are an increasing problem in the developed world. Most existing health services do not have sufficient resources to support long-term individual interventions. The delivery of current disease prevention and management models are not feasible due to their high cost and they do not always reach those who need them. Therefore, new models of prevention and treatment measures based on self-management are needed. Personal health systems including Web-based programs, mobile devices, and other health monitoring tools may be used for self-management of chronic conditions and behavioral change [20].

Internet-based and computer-aided treatments have been shown to be effective in treating a wide range of psychological problems, and have effect sizes (ES) comparable to those found for more traditional types of psychological treatments [21-32]. Seymour and Grove [33] have pointed out that accessibility and acceptability are key issues for further research in addition to effectiveness. To address these issues, Web-based treatment programs can be complemented by mobile and wearable technologies for self-monitoring to best suit the user’s needs and preferences and also to potentially enhance the effect of the intervention. In addition, technology delivered interventions may be complemented by traditional intervention methods such as individual or group face-to-face meetings and phone counseling.

P4Well is a novel CBT- and ACT-based intervention which combines personal health technologies (mobile, Web, and self-monitoring technologies) to an intervention program which is based on group meetings [34,35]. In intervention design, our aim was to combine the cost-effectiveness of the group meetings to a personalized intervention enabled by technology tools. We designed the intervention program content and technology toolkit in parallel, matching them to each other. The P4Well intervention utilizes a variety of technology tools which allow personalization of the intervention methods and feedback. This may increase the acceptance and efficacy of the intervention by giving the users the possibility to choose appropriate self-management tools according to their personal interest.

The objective of this study was to study the feasibility and effectiveness of the developed P4Well intervention among working age males who experienced mild to moderate symptoms of stress and/or depression. We assessed the effects of the intervention using depression, psychological symptoms, and stress as primary outcome measures and compared these effects to a control group without intervention. Secondary outcome measures included quality of life, psychological flexibility, and job strain. Furthermore, we studied the acceptability and usage of the intervention and its components.

## Methods

### Recruitment and Allocation

**Figure 1:**
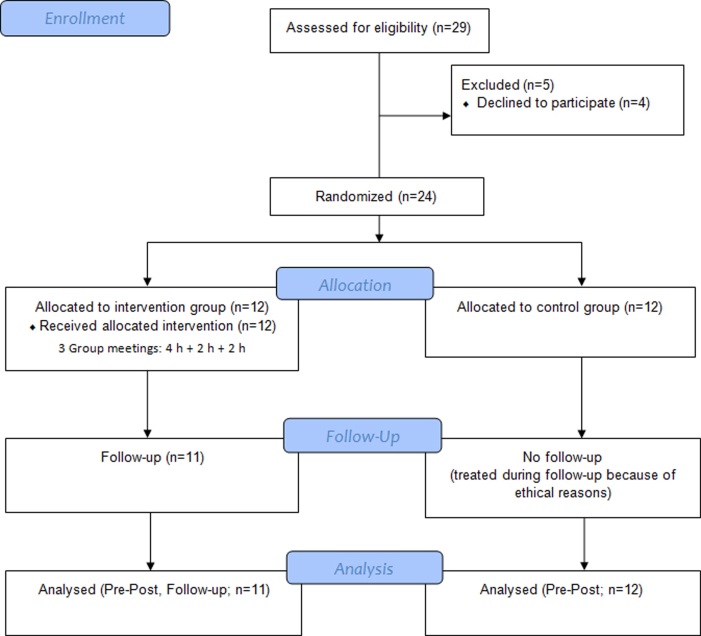
Participant flow chart.

Participants were recruited through an advertisement in a local newspaper, seeking males aged 25 to 45 years old with exhaustion, stress symptoms, or sleeping problems. Other inclusion criteria were full time employment, basic computer skills, and access to Internet. Exclusion criteria included diabetes and simultaneous attendance in other stress management programs. We focused this study on male adults because men have a lower tendency to seek treatment for psychological problems compared to females [36,37]. The psychotherapy clinic of the University of Jyväskylä was contacted by 29 respondents via telephone or email. Before randomization of subjects into the research groups, 4 men dropped out. Since fewer participants responded to the advertisement than expected, we also included respondents older than 45 years of age in the study. The adjusted age range was 28 to 58 years. Of the 25 male participants, we excluded one participant from analysis because he did not fulfill the inclusion criteria (because of age and retirement) and one participant who did not participate in the follow-up measurements. One participant in the intervention group was lost at follow-up (Figure 1). Thus, the total number of participants included in the study was 23. Dropout (n=1) was treated with an intention-to-treat-analysis using the data missing principle of last observation carried forward. We then randomly allocated participants either to the intervention or the waiting list control group (intervention group=11, control group=12). The sample was first divided into pairs based on participants with similar depression scores, measured by Beck’s Depression Inventory (BDI) [38]. Second, the order of pairs was randomized. Third, participants within pairs were randomly assigned either to intervention or control group. Thus, the groups were made equal on the basis of reported depressive symptoms and the researchers generated the randomization. Consent from participants was obtained offline in paper format at pre-measurement. Participants received detailed information about the study procedure and their rights.

The study took place at the psychotherapy clinic of the department of psychology at the University of Jyväskylä, Finland, from January 2009 to October 2009. The Research Ethics Committee of the University of Jyväskylä approved this study. The study was funded by the Finnish Funding Agency for Technology and Innovation (TEKES). The study was not registered in a public trials registry, because the study was a phase 1 small-scale pilot study including no participants with medical or psychiatric diagnosis. The study tested a psychological and technical intervention without any side effects. The funding of the project required that participants with diagnoses should not be included in the study.

### Participants

The mean age of the participants was 47.1 years (SD 4.72) in the intervention group and 39.4 (SD 7.96) in the control group (Table 1). The intervention group was older, *t*_*21*_=2.78, *P*=.011, and had a lower BMI, *t*_*21*_=2.42, *P*=.025, than the control group. The groups did not differ in regard to education, type of work, shift work, or reported depressive symptoms.

**Table 1:** Participant characteristics.

Background variable	Intervention (n=11)	Control (n=12)
Age	47.1 (4.7)	39.4 (8.0)^a^
Body Mass Index	24.4 (3.1)	28.1 (4.2)^a^
Education (yrs)	7.1	7.2
Married (%)	7 (63)	10 (83)
Permanent employment (%)	9 (82)	11 (92)
Fulltime work (%)	10 (91)	12 (100)
Shift work (%)	11 (100)	11 (92)
No physical work (%)	8 (73)	8 (67)
Depressive symptoms (%)^c^	7 (63)	6 (50)
Medication (%)^d^	4 (44)	1 (8)

^a^ *P*=.011

^b^ *P*=.025

^c^ score is 10 or greater in Beck’s Depression Inventory

^d^ use of antidepressants and/or hypnotics

### Intervention

**Figure 2:**
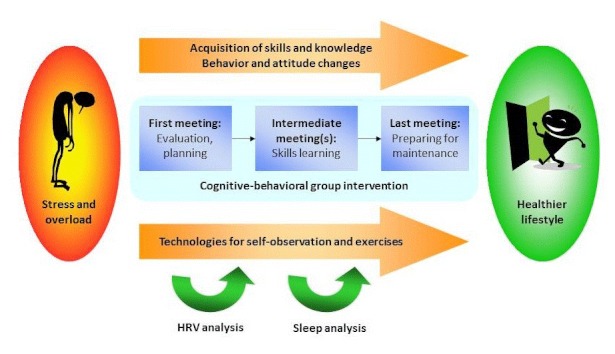
P4well intervention process.

The P4Well intervention integrated different personal health technologies, including a Web portal, mobile phone applications, personal monitoring devices, and analysis software, with a CBT- and ACT-based intervention program which was specifically designed to utilize personal health technologies (Figure 2). The main idea behind the intervention concept was to combine cost-efficiency of group meetings, personalization and self-monitoring capabilities provided by technologies, and, technology use between the group meetings to increase the continuity and impact of the intervention. The intervention program consisted of 3 group meetings held by a psychologist. The main CBT- and ACT-based methods used in the intervention included clarification of personal values, goal setting, self-monitoring, relaxation, mindfulness, and acceptance procedures. Furthermore, regular physical activity was encouraged and emphasized as means for stress reduction, mood elevation, and improved well-being.

Participants placed in the control group did not receive any technical tools or group meetings during the study period. Pre-measurement consisting of self-assessed questionnaires in paper format and heart rate variability recording was done for both the control and intervention groups before the first group meeting. Both groups had an individual assessment meeting during the pre-measurement phase where they received questionnaires and were given a wearable beat-to-beat heart rate (HR) recording device (Suunto Memory Belt, Suunto Ltd, Vantaa, Finland) with instructions to do a 3-day HR variability (HRV) recording. One week after the assessment meeting, the questionnaires and the heart rate belts were collected and analyzed. Feedback was given to both groups by an exercise physiologist. The intervention group received feedback for the HRV recording (1 hour individual discussion of topics concerning stress, sleep and relaxation, and exercise habits) after the first group meeting. Two weeks after the intervention group finished its third and last meeting (ie, after 3 months), both groups were measured for the second time (post-measurement). Follow-up questionnaires were sent to the intervention group 6 months after the intervention ended (intervention group follow-up, n=11). We offered the control group 1 mini-intervention meeting after the post-measurement. Feedback of the recordings for the control group was given during the mini-intervention.

**Figure 3:**
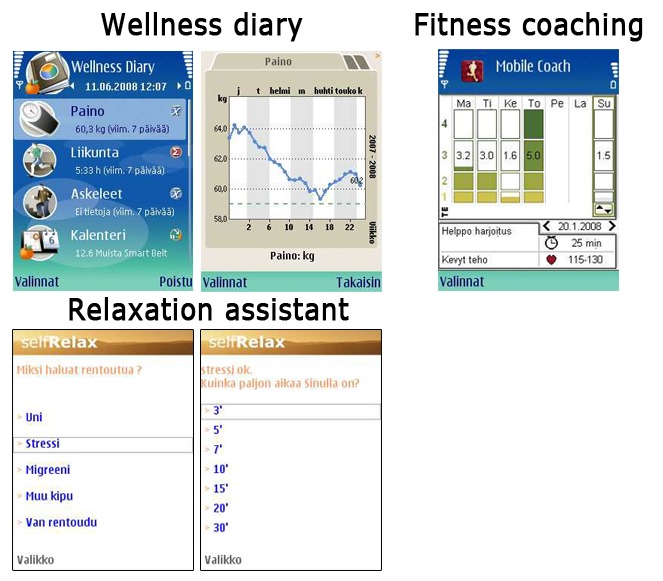
Screenshots of the 3 mobile applications.

The first intervention group meeting was an informative and motivating session that consisted of: (1) measurements (background information questionnaire, technology literacy and attitude questionnaire, and psychological questionnaires), (2) general background information about the P4Well intervention and introduction to the wearable technologies, and (3) the psychological mini-intervention (90 minutes). Participants were provided with credentials to the Web portal, mobile phones (Nokia E51) with preinstalled mobile applications (Wellness diary, Fitness coach, and Relaxation assistant; Figure 3), pedometers, heart rate monitors, and actigraphs. The ACT value analysis method was used to initiate the intervention by motivating behavioral changes in the participants [39,40]. Participants were asked to define their valued directions and goals, as well as actions to accomplish these goals. Additionally, a mindfulness exercise was carried out and participants were instructed to practice mindfulness and relaxation by doing exercises in the Web portal and with a mobile phone application. A self-observation worksheet was presented to encourage participants to begin their self-observations. As a homework assignment, participants were asked to further clarify their personal values and select actions based on these values and to conduct mindfulness exercises. Participants were also asked to start monitoring their sleep with the actigraph. The participants were encouraged but not required to start using one or more mobile applications and begin their self-observations after the group meeting.

The second group meeting (2 hours) was given 4 weeks later. The psychological assessment Web tool in the portal including individual problem analysis was presented and participants were asked to reflect over their situation (eg, stressors in their daily life, sleep, exercise habits, and variables affecting these factors). Participants were encouraged to continue working with the assessment tool at home. The session was ended with a mindfulness exercise. Actigraphs were collected from the participants and analysis reports about sleep and activity were sent to them through the Web portal after the meeting. The participants were encouraged to continue their self-observations with the technology tools.

The third group meeting (2 hours) took place 4 weeks after the second group meeting. The theme of the meeting was acceptance, which involves a willingness to experience all psychological events (thoughts, feelings, and physiological sensations), especially negatively evaluated events, without avoiding, changing, or controlling them [39,40]. Experiential exercises, such as metaphors and exercises related to acceptance, were carried out and discussed. At the end of the group meeting, all of the provided technology tools were collected from the participants. After the meeting, the 3-day HRV recording was repeated, accompanied by an individual stress and recovery analysis. The participants completed the final psychological and user experience questionnaires (sent through mail) 2 weeks after the last group meeting (post-measurement).

### Technology Tools

The personal health technologies that were provided to the participants formed a wellness toolkit from which the participants could choose the most appropriate ones for their needs and preferences. The toolkit included a Web portal, a mobile phone with 3 preinstalled applications, a pedometer and a heart rate monitor. Additionally, the participants wore heart rate belts (Suunto Memory Belt, Suunto Ltd, Vantaa, Finland) for 3 days before and after the intervention period to obtain HRV recordings and actigraphs (Vivago Personal Wellness Manager, Vivago Ltd, Helsinki, Finland) for 4 weeks during the intervention to obtain sleep recordings. Based on these recordings, individual feedback reports were given to the participants. Full details of the P4Well technology toolkit have been described elsewhere and so only a brief outline will be provided here [34,35,41].

**Figure 4:**
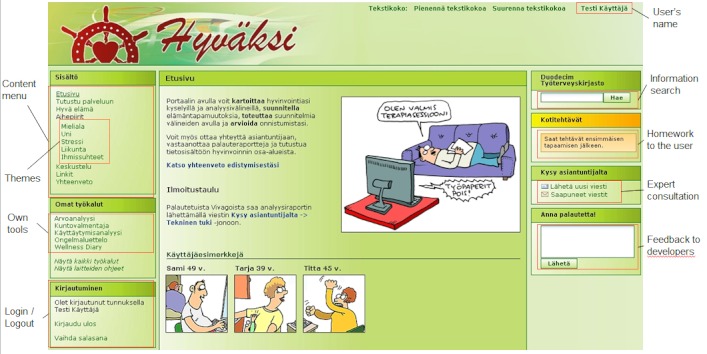
The main screen of the P4Well web portal.

The secured Web portal (Figure 4) provided the participants access to information, exercises, self-assessment and self-reflection tools, Web-based wellness services, peer support, and expert consultation. The content of the portal was divided into modules focusing on different areas of well-being—sleep, exercise, mood, stress and recovery, and good life. The modules consisted of 5 phases: information, evaluation of personal status, planning of lifestyle changes, putting the plans into action, and follow-up. In addition, the portal included a discussion forum and a messaging client for expert consultation. Mobile wellness diary entries were made available through portal interface (Nokia Wellness Diary Connected, Nokia Corp, Espoo, Finland) and access to an adaptive Web-based fitness training program was also included (Firstbeat WebTrainer*,* Firstbeat Technologies Ltd, Jyväskylä, Finland). Finally, the participants could utilize a library of evidence-based health-related information through the portal (Duodecim Health Library, Duodecim Medical Publications Ltd, Helsinki, Finland).

The purpose of the 3 mobile phone applications (Figure 3) was to better integrate wellness management and self-monitoring into the participants’ daily lives. The first application was a mobile wellness diary (Nokia Wellness Diary (WD), Nokia, Espoo, Finland) that could be used to make daily self-observations on wellness related parameters. The second application was a mobile phone version of the fitness training program (Firstbeat Mobile Coach, Firstbeat Technologies Ltd, Jyväskylä, Finland), and the third one was a mobile phone relaxation assistant which included personalized relaxation programs (SelfRelax*,* Relaxline, France).

The participants were encouraged to monitor their physical activity with a heart rate monitor (Suunto Ltd, Vantaa, Finland) or a pedometer (Omron, Kyoto, Japan). Heart rate monitors were primarily meant for participants who were interested in fitness training, whereas pedometers were used to measure and encourage everyday activity. Participants could enter step counts and other exercise parameters manually as daily self-observations into WD.

### Measures

#### Primary Outcome Measures

We measured symptoms of depression using BDI, a widely used 21-item self-report measure of depression [38].

Psychological symptoms were measured using the general symptom index (GSI), which is based on the 90-item symptom checklist (SCL-90). The SCL-90 has been validated for the Finnish population. In a Finnish community sample (n=337) [42], the mean GSI was 0.60 (SD=0.44).

The primary stress measure was the Finnish 15-item version of the Bergen Burnout Indicator (BBI-15) [43], based on the original 25-item Bergen Burnout Indicator [44]. The BBI-15 measures 3 aspects of professional burnout: exhaustion, cynicism, and sense of inadequacy.

#### Secondary Outcome Measures

Quality of life included 5 items: mood, self-rated health, life satisfaction, self-confidence, and working ability. Participants’ perceptions of each item were measured using a visual analogue scale (VAS) from 0 to 100 [45-47].

We measured psychological flexibility and experimental avoidance using the Acceptance and Action Questionnaire-2 (AAQ-2), a 10-item questionnaire that involves both the ability to accept difficult thoughts and feelings as well as to engage in valued activity in their presence (a 7-point Likert-type scale). High scores indicate high psychological flexibility (range 0-70). The AAQ-2 is a revised version of the original AAQ [48].

We measured job strain and over-commitment using the effort-reward imbalance (ERI) questionnaire, which measures extrinsic effort with six items and reward with 11 items. The ratio of effort to reward (ER-ratio) expresses the amount of effort-reward imbalance. High scores indicate high job strain. The ERI questionnaire also includes 6 items that measure over-commitment [49].

### User Experiences and Usage

User experiences were measured post-intervention with a questionnaire about perceived utility and acceptance of each individual technology tool, and the perceived usefulness of different intervention components. The perceived utility of the intervention as a whole was assessed with questions about perceived benefits from participation in the study. Usage logs were collected from the portal and the mobile applications after the end of the intervention. For pedometers and heart rate monitors, usage frequency was assessed through the post-intervention questionnaire. Participants were defined as active users of a given tool if they had used it during at least half of the study weeks (based on log data) or reported having used it at least weekly (questionnaire data).

### Statistical Analyses

We performed statistical analyses using SPSS 15.0 for Windows (SPSS, Inc, Chicago, IL). A repeated-measures ANOVA evaluated the intervention effect with group (intervention vs control) as the between-subjects factor, and pre- and post-measurements as the within-subject factor. When analyzing pre-, post-, and follow-up measurements of the intervention group, a repeated-measures ANOVA was used. The level of statistical significance was set at *P*<.05; however, due to the small sample size, we took into account interactions where *P*<.10. The ES, measured by Cohen's d, were calculated to measure clinically significant between group differences and within group changes. We calculated the post-treatment between-group ES by dividing the difference between the treatment mean and the control mean with the pooled standard deviation of the two conditions. The within-group ES was calculated by dividing the mean change from pre- to post- with the pre-treatment SD and the mean change from pre- to follow-up with the pre-treatment SD [50,51]. Between-group ESs of 0.2, 0.5, and 0.8 were considered small, medium, and large, respectively. Within-group ESs of 0.5, 0.8, and 1.1 were treated likewise [7,52].

## Results

### Acceptance and Usage

All participants in the intervention group stated that their well-being had improved as a result of the intervention. The most common benefits the participants reported included increased willingness to improve personal well-being (8/11, 73%), decreased level of stress (6/11, 55%), and increased amount of exercise (5/11, 45%). The most useful intervention components were considered to be measurements and feedback (10/11, 91%), personal monitoring devices (9/11, 82%), group meetings (8/11, 73%,), and mobile applications (6/11, 55%).

All participants tried at least 3 out of 6 available tools (mean 4.7, range 3-6) and 10/11 participants used at least 1 of the tools (mean 1.9, range 1-4) actively. Each tool had at least 1 active user (Table 2). The mobile relaxation application had the highest number of active users. Pedometer was ranked as the easiest and most personally suitable. Heart rate monitor was rated as the most useful and difficult to use.

**Table 2:** Usage and user experiences of technology tools.

	Web portal	Wellness diary	Exercise coaching	Relaxation application	Pedometer	Heart rate monitor
Active users (n)	3	1	4	5	4	4
Easy to use^a^	6	5	5	8	11	0
Useful^a^	5	5	7	6	7	8
Personally suitable^a^	5	3	7	6	8	7
Motivating^a^	3	5	6	7	6	6

^a^Values are the numbers of users who agreed or strongly agreed with the statement.

### Efficacy

Depressive symptoms, as measured by BDI, decreased more in the intervention group compared to the control group (Table 3). There was a marginally significant group by time interaction effect for BDI (*P*=.072). The mean BDI value decreased with more than 8 scores (CI 4.92-11.99) in the treatment group compared to four scores (CI 0.62-7.38) in the control group. We found a medium ES (d=0.57) between groups in favor of the intervention group. Participants maintained positive changes at the 6-month follow-up. We found a significant within-group effect over time for the intervention group (*P*=.001): both post- and follow-up measurements were significantly lower compared to the BDI pre-measurement. Pre- to follow-up BDI measurements indicated a large within-group ES (d=1.11). An analysis of the number of the participants who reported depressive symptoms at pre-, post-, and follow-up measurements also suggested that the intervention had a positive effect on mood. At the beginning of the study, 64% (7/11) of the participants in the intervention group and 50% (6/12) in the control group reported at least mild depression (a BDI of at least 10). Only 9% (1/11) reported depressive symptoms in the intervention group after the intervention ended. In the control group, 50% (6/12) were still depressed at post-measurement. At follow-up, only 1 person (9%) in the intervention group reported BDI values greater than 10.

Psychological symptoms (SCL-90) decreased in the intervention group but remained at the same level in the control group (Table 3). We found a marginally significant group by time interaction effect in psychological symptoms (*P*=.053). The between-group ES was small (d=0.39). The within-group ES from pre- to follow-up measurement was medium (d=1.07). Again, we found a significant within-group effect for the intervention group—both the post- and follow-up measurements were significantly lower compared to pre-measurement scores. A significant group by time interaction effect was found for health (*P*=.008) and working ability (*P*=.016). The between- and within-group ESs were small for both health (d=0.38 and 0.56, respectively) and working ability (d=0.21 and 0.60, respectively). Furthermore, for these variables we found a significant within-group effect in the intervention group. Thus, health was rated higher after treatment, and participants estimated their working ability to be higher at follow-up compared to the beginning of the treatment. As we can see from Table 3, there was some indication that life satisfaction increased from pre-measurement to follow-up, as well.

**Table 3:** Psychological symptoms and life quality for the intervention and control group.

		Pre Mean (SD)	Post Mean (SD)	95% CI for the difference		Follow-up Mean (SD)	Pre-Post group x time	Intervention within effect
				Lower	Upper			
**Depression BDI**								
	Intervention	14.64	6.18	4.92	11.99	6.18	F_1,21_=3.59	F_2,20_=17.45
		(7.61)	(3.31)			(3.28)	*P*=.072	*P*=.001
	Control	13.33	9.33	0.62	7.38	-	d=0.57	d=1.11
		(9.24)	(7.10)					
**Symptom SCL**								
	Intervention	0.64	0.40	0.11	0.37	0.35	F_1,21_=4.22	F_2,20_=10.28
		(0.27)	(0.18)			(0.18)	*P*=.053	*P*=.001
	Control	0.57	0.51	-0.07	0.18	-	d=0.39	d=1.07
		(0.30)	(0.36)					
**Psych Flex AAQ**								
	Intervention	52.46	55.73	-7.95	1.40	55.45	F_1,21_=1.74	F_2,20_=0.93
		(10.00)	(6.25)			(7.26)	*P*=.201	*P*=.41
	Control	54.50	53.67	-3.64	5.31	-	d=0.25	d=0.30
		(7.82)	(9.60)					
**Life Satisfaction**								
	Intervention	59.91	66.09	-14.78	2.42	69.27	F_1,21_=0.04	F_2,20_=5.68
		(15.55)	(10.51)			(13.45)	*P*=.838	*P*=.01
	Control	59.92	64.92	-13.23	3.23	-	d=0.09	d=0.60
		(17.29)	(15.50)					
**Self-rated Health**								
	Intervention	63.27	74.91	-18.89	-4.38	71.09	F_1,21_=8.57	F_2,20_=5.18
		(13.86)	(11.64)			(15.41)	*P*=.008	*P*=.02
	Control	72.42	69.92	-4.44	9.44	-	d=0.38	D=0.56
		(10.26)	(14.49)					
**Mood**								
	Intervention	60.27	66.82	-14.38	1.29	66.09	F_1,21_=0.08	F_2,20_=1.50
		(17.35)	(8.34)			(13.32)	*P=*.783	*P*=.25
	Control	57.08	65.08	-15.50	-0.50	-	d=0.15	d=0.34
		(16.51)	(14.18)					
**Self-Confidence**								
	Intervention	63.55	70.27	-15.80	2.34	74.73	F_1,21_=0.07	F_2,20_=2.49
		(15.63)	(15.85)			(16.41)	*P*=.788	*P*=.11
	Control	69.58	74.67	-13.77	3.6	-	d=0.34	d=0.72
		(13.76)	(9.21)					
**Working Ability**								
	Intervention	64.36	74.00	-16.93	-2.34	75.45	F_1,21_=6.86	F_2,20_=5.48
		(20.25)	(15.93)			(13.48)	*P*=.016	*P*=.01
	Control	74.00	70.92	-3.9	10.07	-	d=0.21	d=0.60
		(7.79)	(12.91)					

We did not observe a significant group by time interaction for burnout (Table 4). However, the burnout scores decreased from pre-measurement to follow-up and showed a medium ES (d=0.91). In the intervention group we found a significant within-group effect on cynicism, although there was no significant group by time interaction. However, we obtained a medium (between group) ES for cynicism. There were marginally significant interaction effects on effort (*P*=.07) and over-commitment (*P*=.08). The scores for over-commitment were lower at follow-up compared to the beginning of treatment. The within-group ES from pre-measurement to follow-up was small for over-commitment (d=0.61).

**Table 4:** Burnout and stress for the intervention and control group.

		Pre Mean (SD)	Post Mean (SD)	95% CI for the difference		Follow-up Mean (SD)	Pre-Post group x time	Intervention within effect
				Lower	Upper			
**Burnout**								
	Intervention	3.52	3.03	0.16	0.82	2.88	F_1,21_=1.02	F_2,20_=6.67
		(0.70)	(0.83)			(0.73)	*P*=.32	*P*=.006
	Control	3.64	3.38	-0.5	0.59	-	d=0.47	d=0.91
		(0.70)	(0.64)					
**Exhaustion**								
	Intervention	3.96	3.67	-0.09	0.67	3.42	F_1,21_=0.01	F_2,20_=3.08
		(1.10)	(1.15)			(1.06)	*P*=.93	*P*=.07
	Control	4.13	3.87	-0.10	0.63	-	d=0.20	d=0.49
		(0.67)	(0.84)					
**Cynicism**								
	Intervention	3.15	2.42	0.32	1.14	2.22	F_1,21_=2.63	F_2,20_=10.94
		(0.93)	(0.96)			(0.75)	*P*=.12	*P*=.001
	Control	3.22	2.93	-0.11	0.68	-	d=0.60	d=1.00
		(0.78)	(0.73)					
**Sense of Inadequancy**								
	Intervention	3.46	3.00	-0.1	0.92	3.00	F_1,21_=0.44	F_2,20_=2.04
		(1.10)	(0.96)			(1.02)	*P*=.51	*P*=.16
	Control	3.58	3.33	-0.19	0.69	-	d=0.35	d=0.41
		(1.04)	(0.95)					
**Effort**								
	Intervention	3.39	3.21	-0.9	0.45	3.04	F_1,21_=3.74	F_2,18_=2.08
		(0.82)	(0.95)			(0.90)	*P*=.07	*P*=.15
	Control	3.26	3.43	-0.43	0.09	-	d=0.36	d=0.41
		(0.66)	(0.40)					
**Reward**								
	Intervention	3.69	3.88	-0.56	0.18	4.18	F_1,21_=0.59	F_2,18_=2.21
		(0.68)	(0.95)			(0.48)	*P*=.45	*P*=.14
	Control	3.64	4.02	-0.73	-0.03	-	d=0.04	d=0.62
		(0.89)	(0.72)					
**Effort-reward imbalance**								
	Intervention	0.96	0.91	-0.10	0.19	0.74	F_1,21_=0.02	F_2,18_=2.43
		(0.29)	(0.43)			(0.25)	*P*=.89	*P*=.12
	Control	0.94	0.89	-0.08	0.19	-	d=0.13	d=0.69
		(0.27)	(0.23)					
**Over commitment**								
	Intervention	2.92	2.64	0.05	0.53	2.48	F_1,20_=3.53	F_2,18_=4.03
		(0.64)	(0.61)			(0.57)	*P*=.08	*P*=.04
	Control	2.79	2.80	-0.25	0.22	-	d=0.44	d=0.61
		(0.53)	(0.44)					

In the group intervention, the amount of therapist face-to-face contact time was 8 hours (4 + 2 + 2), totalling 480 minutes (including measurements). Thus, the therapist contact time used for each participant during the intervention was 44 minutes.

## Discussion

The objective of this study was to assess the feasibility of the P4Well intervention in the target population of working-age adults who experience mild psychological and stress-related symptoms. Our results confirm that the intervention was acceptable and personal health technologies were actively used by the participants. The results also suggest that the intervention had a positive effect on our primary outcome measures (depressive and psychological symptoms) as well as on self-rated health and working ability. The intervention was also cost-effective. The total professional time used during the active intervention period was less than 1 hour per person.

Before the intervention, the majority of participants reported symptoms of depression; after the intervention only 1 reported symptoms of depression. The intervention group’s within-group ES (measuring clinical significance) from pre-measurement to the follow-up was large and the between-group post-treatment ES was medium. These effects are in line with other studies investigating the effects of cognitive-behavioral methods. Meta-analysis from Gloaguen et al [53] found that the between-group ES between CBT and controls was typically d=0.82. In our study, the ES was somewhat smaller (d=0.57), however the ES was at least the same or larger compared to groups taking anti-depressant medications. Our data also indicated that the intervention might have positive effects on burnout symptoms: participants’ BBI-15 scores were lower at the 6-month follow-up compared to the beginning of the treatment. Moreover, the results suggest that there were positive effects on cynicism and over-commitment related to recovery from stress and burnout.

Participants perceived the intervention as beneficial and useful, and reported reduced amount of stress, increased physical activity, and greater motivation to improve their well-being. Almost everyone took some of the technology tools into active use and each tool was considered useful, motivating, and personally suitable by several participants. These results suggest that offering several tools and techniques to support changes in multiple behaviors may be a promising approach in interventions that address psychological problems. Most interventions to this date have tailored their content to individual needs, but few have used multiple applications or delivery channels that could be freely chosen by participants based on their preferences. There is a wealth of applications and devices available for self-monitoring of stress, mood, physical activity and sleep, and for relaxation and mindfulness skills training. Nevertheless, individuals struggling with psychological problems and stress may not be aware of the existence or usefulness of these tools. Based on the wide variety of reasons and behavioral treatment options for psychological problems, intervention outcomes and adherence may be improved by matching and recommending specific applications and/or devices to different needs and preferences of participants [54]. An intervention program should be designed with careful consideration of appropriate technology tools that best serve the purposes of the intervention.

Even though personal monitoring devices and mobile applications were received favorably and used actively, human contact was still highly valued. Personal feedback and advice based on physiological measurements was considered the most useful component of the intervention, and group meetings were also appreciated. Peer and counselor support may be crucial factors that increase participant engagement and motivation in technology-based interventions [55]. Interestingly, measurements and personal monitoring devices were evaluated as useful as group meetings. Technology can facilitate remote consultation regardless of time and place, hence reducing the costs and widening the reach and accessibility of interventions. Furthermore, leveraging technology tools in intervention delivery optimizes the use of professionals’ time, since it allows participants to complete routine exercises and tasks independently with automated and personalized guidance.

There is an acute need to improve people’s psychological well-being, especially depression and various stress-related problems. These problems are widespread and can lead to long-term absenteeism and work disability, which include a significant economic burden [4,56]. Our data suggest that it is possible to positively affect psychological well-being by using interventions that combine face-to-face meetings and technology. Our intervention may be a noteworthy tool for self-management, health-related prevention and general well-being in health care settings. Prevention and early intervention based on self-management are especially important given that healthcare resources are limited. In addition, our intervention offers considerable flexibility, and requires only little professional guidance. In accordance with earlier studies that have investigated the combination of technology and multimodal intervention methods to promote health, our results suggest that interventions using technologies can extend the reach of preventive care to many people at a relatively low cost [57-61]. Our findings are also in line with other studies that show positive effects using Web-based stress management approaches [11, 62].

This study had several limitations. First of all, the number of participants was small and therefore the statistical power of the study is weak. This lack of power affects our ability to detect differences between the intervention and control group as well as our ability to generalize the results. Also, most participants reported a small number of psychological problems at the beginning of the study. Thus, the possibility for improvement was small (eg, for AAQ, BDI and SCL-90) suggesting that other measurements may have been more appropriate for observing the changes. The control group showed also some improvement that was possibly due to assessment procedures at the beginning. The effectiveness and the acceptability of this intervention need also to be investigated in other populations reporting more severe problems. Furthermore, longer follow-up periods may be needed to ensure the sustainability of the effect. Overall, because all the participants in this study were male, our results can be generalized to only middle-aged men who seek help for stress-related problems and mild to moderate depression. Additionally, participants were provided several technology tools within a short period of time, which caused cognitive load that may have hindered participants’ capability and motivation to discover personally suitable tools. Some tools also had usability problems, data entered in one application was not synchronized to others, and most of self-monitoring was done manually. Since the time the study was conducted, there have been considerable advances especially in smartphone technology, which would allow a more integrated and usable technology toolkit.

In conclusion, this study supports the idea that personal health technologies, when combined with a brief psychological group intervention program, may have a positive impact on mild psychological problems and stress-related symptoms. Our intervention provides a potential solution to the demand for accessible and affordable empirically-supported psychological treatments [63]. Our approach is potentially cost-efficient, flexible and accessible, and may help people to prevent and manage stress-related problems and to adopt a healthier lifestyle. However, due to the limitations in the design and procedure, our results may be spurious, and must be interpreted with caution. Furthermore, because the intervention included several components and it was not possible to control all of them within our design, we cannot rule out that these effects were caused by the group sessions alone. Although there are several limitations in this study, this intervention nevertheless shows promising effects. Future studies need to investigate the effectiveness, benefits, and possible problems of psychological interventions which incorporate new technologies. Our aim is to enhance and simplify the presented concept, and evaluate it in a larger, more comprehensive research study using mobile technology. A fully powered RCT using partly the same concept is under way. In the ongoing study, a brief group ACT-based intervention is compared with an ACT-based mobile intervention.

## Multimedia Appendix 1

CONSORT-eHealth Checklist V1.6.1 [64].

## Abbreviations

AAQ-2: acceptance and action questionnaire
ACT: acceptance and commitment therapy
BBI-15: Bergen Burnout Indicator
BMI: body mass index
CBT: cognitive behavorial therapies
ER-ratio: effort to reward ratio
ERI: effort-reward imbalance
ES: effect sizes
GSI: general symptom index
HR: heart rate
HRV: heart rate variability
SCL-90: 90-item symptom checklist
TEKES: Finnish Funding Agency for Technology and Innovation
VAS: visual analogue scale
WD: wellness diary
