# Supplementary material for: Feasibility of a Personal Health Technology-Based Psychological Intervention for Men with Stress and Mood Problems: Randomized Controlled Pilot Trial
Source: JMIR Res Protoc. 2013 Jan 9;2(1):e1. doi: 10.2196/resprot.2389 (PMC3628150; doi:10.2196/resprot.2389)
Supplement: Supplementary file 1 [file resprot_v2i1e1_app1.pdf]

CONSORT-EHEALTH (V 1.6.1) - Submission/Publication Form

The CONSORT-EHEALTH checklist is intended for authors of randomized trials evaluating web-based and Internet-based applications/interventions, including mobile interventions, electronic games (incl multiplayer games), social media, certain telehealth applications, and other interactive and/or networked electronic applications. Some of the items (e.g. all subitems under item 5 - description of the intervention) may also be applicable for other study designs.

The goal of the CONSORT EHEALTH checklist and guideline is to be
a) a guide for reporting for authors of RCTs,
b) to form a basis for appraisal of an ehealth trial (in terms of validity)

CONSORT-EHEALTH items/subitems are MANDATORY reporting items for studies published in the Journal of Medical Internet Research and other journals / scientific societies endorsing the checklist.

Items numbered 1., 2., 3., 4a., 4b etc are original CONSORT or CONSORT-NPT (non-pharmacologic treatment) items.
Items with Roman numerals (i., ii, iii, iv etc.) are CONSORT-EHEALTH extensions/clarifications.

As the CONSORT-EHEALTH checklist is still considered in a formative stage, we would ask that you also RATE ON A SCALE OF 1-5 how important/useful you feel each item is FOR THE PURPOSE OF THE CHECKLIST and reporting guideline (optional).

Mandatory reporting items are marked with a red *.
In the textboxes, either copy & paste the relevant sections from your manuscript into this form - please include any quotes from your manuscript in QUOTATION MARKS,
or answer directly by providing additional information not in the manuscript, or elaborating on why the item was not relevant for this study.

YOUR ANSWERS WILL BE PUBLISHED AS A SUPPLEMENTARY FILE TO YOUR PUBLICATION IN JMIR AND ARE CONSIDERED PART OF YOUR PUBLICATION (IF ACCEPTED).
Please fill in these questions diligently. Information will not be copyedited, so please use proper spelling and grammar, use correct capitalization, and avoid abbreviations.

DO NOT FORGET TO SAVE AS PDF _AND_ CLICK THE SUBMIT BUTTON SO YOUR ANSWERS ARE IN OUR DATABASE !!!
*Pakollinen

Your name *
First Last

Primary Affiliation (short), City, Country *
University of Toronto, Toronto, Canada

Your e-mail address *
abc@gmail.com

Title of your manuscript *

Provide the (draft) title of your manuscript.

Feasibility of a Personal Health Technology -based Intervention for Men with Stress and Mood Problems: Randomized Controlled Trial

Article Preparation Status/Stage *

At which stage in your article preparation are you currently (at the time you fill in this form)

- not submitted yet - in early draft status
- not submitted yet - in late draft status, just before submission
- submitted to a journal but not reviewed yet
- submitted to a journal and after receiving initial reviewer comments
- submitted to a journal and accepted, but not published yet
- published
- Muu:

Journal *

If you already know where you will submit this paper (or if it is already submitted), please provide the journal name (if it is not JMIR, provide the journal name under "other")

- not submitted yet / unclear where I will submit this
- Journal of Medical Internet Research (JMIR)
- Muu:

Manuscript tracking number *

If this is a JMIR submission, please provide the manuscript tracking number under "other" (The ms tracking number can be found in the submission acknowledgement email, or when you login as author in JMIR. If the paper is already published in JMIR, then the ms tracking number is the four-digit number at the end of the DOI, to be found at the bottom of each published article in JMIR)

- no ms number (yet) / not (yet) submitted to / published in JMIR
- Muu:

TITLE AND ABSTRACT

1a) TITLE: Identification as a randomized trial in the title

1a) Does your paper address CONSORT item 1a? *
I.e does the title contain the phrase "Randomized Controlled Trial"? (if not, explain the reason under "other")

- ( ) yes
- ( ) Muu: [ ]

1a-i) Identify the mode of delivery in the title
Identify the mode of delivery. Preferably use “web-based” and/or “mobile” and/or “electronic game” in the title. Avoid ambiguous terms like “online”, “virtual”, “interactive”. Use “Internet-based” only if Intervention includes non-web-based Internet components (e.g. email), use “computer-based” or “electronic” only if offline products are used. Use “virtual” only in the context of “virtual reality” (3-D worlds). Use “online” only in the context of “online support groups”. Complement or substitute product names with broader terms for the class of products (such as “mobile” or “smart phone” instead of “iphone”), especially if the application runs on different platforms.

	1	2	3	4	5	
subitem not at all important	( )	( )	( )	( )	( )	essential

Does your paper address subitem 1a-i? *
Copy and paste relevant sections from manuscript title (include quotes in quotation marks "like this" to indicate direct quotes from your manuscript), or elaborate on this item by providing additional information not in the ms, or briefly explain why the item is not applicable/relevant for your study

"A Personal Health Technology -based Intervention"

1a-ii) Non-web-based components or important co-interventions in title
Mention non-web-based components or important co-interventions in title, if any (e.g., “with telephone support”).

	1	2	3	4	5	
subitem not at all important	( )	( )	( )	( )	( )	essential

Does your paper address subitem 1a-ii?
Copy and paste relevant sections from manuscript title (include quotes in quotation marks "like this" to indicate direct quotes from your manuscript), or elaborate on this item by providing additional information not in the ms, or briefly explain why the item is not applicable/relevant for your study

"psychological Intervention"

1a-iii) Primary condition or target group in the title

Mention primary condition or target group in the title, if any (e.g., “for children with Type I Diabetes”) Example: A Web-based and Mobile Intervention with Telephone Support for Children with Type I Diabetes: Randomized Controlled Trial

	1	2	3	4	5	
subitem not at all important	○	○	○	○	○	essential

Does your paper address subitem 1a-iii? *

Copy and paste relevant sections from manuscript title (include quotes in quotation marks "like this" to indicate direct quotes from your manuscript), or elaborate on this item by providing additional information not in the ms, or briefly explain why the item is not applicable/relevant for your study

"for men"

1b) ABSTRACT: Structured summary of trial design, methods, results, and conclusions

NPT extension: Description of experimental treatment, comparator, care providers, centers, and blinding status.

1b-i) Key features/functionalities/components of the intervention and comparator in the METHODS section of the ABSTRACT

Mention key features/functionalities/components of the intervention and comparator in the abstract. If possible, also mention theories and principles used for designing the site. Keep in mind the needs of systematic reviewers and indexers by including important synonyms. (Note: Only report in the abstract what the main paper is reporting. If this information is missing from the main body of text, consider adding it)

	1	2	3	4	5	
subitem not at all important	○	○	○	○	○	essential

Does your paper address subitem 1b-i? *

Copy and paste relevant sections from the manuscript abstract (include quotes in quotation marks "like this" to indicate direct quotes from your manuscript), or elaborate on this item by providing additional information not in the ms, or briefly explain why the item is not applicable/relevant for your study

"P4Well is a novel intervention which combines modern psychotherapy (Cognitive Behavioral Therapy and Acceptance and Commitment Therapy) with personal health technologies to deliver the intervention via multiple channels (group meetings, Internet, mobile, and personal devices)."

1b-ii) Level of human involvement in the METHODS section of the ABSTRACT

Clarify the level of human involvement in the abstract, e.g., use phrases like “fully automated” vs. “therapist/nurse/care provider/physician-assisted” (mention number and expertise of providers involved, if any). (Note: Only report in the abstract what the main paper is reporting. If this information is missing from the main body of text, consider adding it)

	1	2	3	4	5	
subitem not at all important	○	○	○	○	○	essential

Does your paper address subitem 1b-ii?

Copy and paste relevant sections from the manuscript abstract (include quotes in quotation marks "like this" to indicate direct quotes from your manuscript), or elaborate on this item by providing additional information not in the ms, or briefly explain why the item is not applicable/relevant for your study

"In addition to personal health technologies for self-assessment, the intervention consisted of three psychologist-assisted group meetings."

1b-iii) Open vs. closed, web-based (self-assessment) vs. face-to-face assessments in the METHODS section of the ABSTRACT

Mention how participants were recruited (online vs. offline), e.g., from an open access website or from a clinic or a closed online user group (closed usergroup trial), and clarify if this was a purely web-based trial, or there were face-to-face components (as part of the intervention or for assessment). Clearly say if outcomes were self-assessed through questionnaires (as common in web-based trials). Note: In traditional offline trials, an open trial (open-label trial) is a type of clinical trial in which both the researchers and participants know which treatment is being administered. To avoid confusion, use “blinded” or “unblinded” to indicated the level of blinding instead of “open”, as “open” in web-based trials usually refers to “open access” (i.e. participants can self-enrol). (Note: Only report in the abstract what the main paper is reporting. If this information is missing from the main body of text, consider adding it)

	1	2	3	4	5	
subitem not at all important	○	○	○	○	○	essential

Does your paper address subitem 1b-iii?

Copy and paste relevant sections from the manuscript abstract (include quotes in quotation marks "like this" to indicate direct quotes from your manuscript), or elaborate on this item by providing additional information not in the ms, or briefly explain why the item is not applicable/relevant for your study

Participants were recruited offline using a newspaper advertisement. There were both face-to-face component and personal health technology involved:
"In addition to personal health technologies for self-assessment, the intervention consisted of three psychologist-assisted group meetings."

"Self-assessed psychological measures through questionnaires were collected offline pre- and post-intervention, and 6 months after the intervention for the intervention group. Acceptance and usage of technology tools were measured with user experience questionnaires and usage logs."

1b-iv) RESULTS section in abstract must contain use data

Report number of participants enrolled/assessed in each group, the use/uptake of the intervention (e.g., attrition/adherence metrics, use over time, number of logins etc.), in addition to primary/secondary outcomes. (Note: Only report in the abstract what the main paper is reporting. If this information is missing from the main body of text, consider adding it)

	1	2	3	4	5	
subitem not at all important	○	○	○	○	○	essential

Does your paper address subitem 1b-iv?

Copy and paste relevant sections from the manuscript abstract (include quotes in quotation marks "like this" to indicate direct quotes from your manuscript), or elaborate on this item by providing additional information not in the ms, or briefly explain why the item is not applicable/relevant for your study

"A total of 11 participants were treated in the intervention group and 12 participants did not receive any intervention (control group). Depressive and psychological symptoms decreased and self-rated health and working ability increased. All technology tools had active users and 10/11 participants used at least one tool actively. Physiological measurements with personal feedback were considered the most useful intervention component."

1b-v) CONCLUSIONS/DISCUSSION in abstract for negative trials

Conclusions/Discussions in abstract for negative trials: Discuss the primary outcome - if the trial is negative (primary outcome not changed), and the intervention was not used, discuss whether negative results are attributable to lack of uptake and discuss reasons. (Note: Only report in the abstract what the main paper is reporting. If this information is missing from the main body of text, consider adding it)

	1	2	3	4	5	
subitem not at all important	○	○	○	○	○	essential

Does your paper address subitem 1b-v?

Copy and paste relevant sections from the manuscript abstract (include quotes in quotation marks "like this" to indicate direct quotes from your manuscript), or elaborate on this item by providing additional information not in the ms, or briefly explain why the item is not applicable/relevant for

your study

No

INTRODUCTION

2a) In INTRODUCTION: Scientific background and explanation of rationale

2a-i) Problem and the type of system/solution

Describe the problem and the type of system/solution that is object of the study: intended as stand-alone intervention vs. incorporated in broader health care program? Intended for a particular patient population? Goals of the intervention, e.g., being more cost-effective to other interventions, replace or complement other solutions? (Note: Details about the intervention are provided in “Methods” under 5)

	1	2	3	4	5	
subitem not at all important	○	○	○	○	○	essential

Does your paper address subitem 2a-i? *

Copy and paste relevant sections from the manuscript (include quotes in quotation marks "like this" to indicate direct quotes from your manuscript), or elaborate on this item by providing additional information not in the ms, or briefly explain why the item is not applicable/relevant for your study

"Lifestyle-related chronic conditions are an increasing problem in the developed world. Most existing health services do not have sufficient resources to support long-term individual interventions. Our aim was to combine the cost-effectiveness of the group meetings to a personalized intervention enabled by technology tools."

2a-ii) Scientific background, rationale: What is known about the (type of) system

Scientific background, rationale: What is known about the (type of) system that is the object of the study (be sure to discuss the use of similar systems for other conditions/diagnoses, if appropiate), motivation for the study, i.e. what are the reasons for and what is the context for this specific study, from which stakeholder viewpoint is the study performed, potential impact of findings [2]. Briefly justify the choice of the comparator.

	1	2	3	4	5	
subitem not at all important	○	○	○	○	○	essential

Does your paper address subitem 2a-ii? *

Copy and paste relevant sections from the manuscript (include quotes in quotation marks "like this" to indicate direct quotes from your manuscript), or elaborate on this item by providing additional information not in the ms, or briefly explain why the item is not applicable/relevant for your study

"Besides traditional CBT methods, research suggests that stress management interventions based on Acceptance and Commitment Therapy (ACT) have a positive impact on employees' psychological health, wellbeing and stress management skills"
"...web-based treatment programs can be complemented by mobile and wearable technologies for self-monitoring to best suit the user's needs and preferences and also to potentially enhance the effect of the intervention."
"P4Well is a novel CBT- and ACT-based intervention which combines personal health technologies (mobile, web and self-monitoring technologies) to an intervention program which is based on group meetings"

2b) In INTRODUCTION: Specific objectives or hypotheses

Does your paper address CONSORT subitem 2b? *

Copy and paste relevant sections from the manuscript (include quotes in quotation marks "like this" to indicate direct quotes from your manuscript), or elaborate on this item by providing additional information not in the ms, or briefly explain why the item is not applicable/relevant for your study

"The objective of this study was to study the feasibility and effectiveness of the developed P4Well intervention among male, working-age citizens who experienced mild to moderate symptoms of stress and/or depression. We assessed the effects of the intervention using depression, psychological symptoms and stress as primary outcome measures and compared these effects to a control group without intervention. Secondary outcome measures included quality of life, psychological flexibility and job strain. Furthermore, we studied the acceptability and usage of the intervention and its components. ."

METHODS

3a) Description of trial design (such as parallel, factorial) including allocation ratio

Does your paper address CONSORT subitem 3a? *

Copy and paste relevant sections from the manuscript (include quotes in quotation marks "like this" to indicate direct quotes from your manuscript), or elaborate on this item by providing

additional information not in the ms, or briefly explain why the item is not applicable/relevant for your study

"Participants were recruited through an advertisement in a local newspaper seeking males aged 25- to 45-years-old with exhaustion, stress symptoms or sleeping problems. Other inclusion criteria were full time employment, basic computer skills and access to Internet. Exclusion criteria included diabetes and simultaneous attendance in another stress management program. We focused this study on male adults because men have a lower tendency to seek treatment for psychological problems compared to females. Twenty-nine respondents contacted the psychotherapy clinic of the University of Jyväskylä via telephone or E-mail. Four men dropped out before randomizing the subjects to the research groups. Of the 25 male participants, we excluded one participant from analysis because of not fulfilling the inclusion criteria (because of age and retirement) and one participant who did not participate in the follow-up measurements. One participant in the intervention group was lost at follow-up (see flow diagram).Thus, the total number of participants included in the study was 23." We divided the participants into two categories: those reporting depressive symptoms (score ≥ 10; at least mild depression) and those not reporting depressive symptoms (score < 10; no depression), as measured by Beck's Depression Inventory BDI. We then randomly allocated these samples either to the intervention or the waiting-list control group (intervention group=11, control group=12). Thus, the groups were made equal on the basis of reported depressive symptoms.

3b) Important changes to methods after trial commencement (such as eligibility criteria), with reasons

Does your paper address CONSORT subitem 3b? *

Copy and paste relevant sections from the manuscript (include quotes in quotation marks "like this" to indicate direct quotes from your manuscript), or elaborate on this item by providing additional information not in the ms, or briefly explain why the item is not applicable/relevant for your study

"Because the number of eligible participants was low, we also included respondents older than 45 years of age in the study. The adjusted age range was 28 to 58 years."

3b-i) Bug fixes, Downtimes, Content Changes

Bug fixes, Downtimes, Content Changes: ehealth systems are often dynamic systems. A description of changes to methods therefore also includes important changes made on the intervention or comparator during the trial (e.g., major bug fixes or changes in the functionality or content) (5-iii) and other “unexpected events” that may have influenced study design such as staff changes, system failures/downtimes, etc. [2].

	1	2	3	4	5	
subitem not at all important	○	○	○	○	○	essential

Does your paper address subitem 3b-i?

Copy and paste relevant sections from the manuscript (include quotes in quotation marks "like this" to indicate direct quotes from your manuscript), or elaborate on this item by providing additional information not in the ms, or briefly explain why the item is not applicable/relevant for your study

We didn't experience any unexpected events.

4a) Eligibility criteria for participants

Does your paper address CONSORT subitem 4a? *

Copy and paste relevant sections from the manuscript (include quotes in quotation marks "like this" to indicate direct quotes from your manuscript), or elaborate on this item by providing additional information not in the ms, or briefly explain why the item is not applicable/relevant for your study

"Participants were recruited through an advertisement in a local newspaper seeking males aged 25- to 45-years-old with exhaustion, stress symptoms or sleeping problems. Other inclusion criteria were full time employment, basic computer skills and access to Internet. Exclusion criteria included diabetes and simultaneous attendance in another stress management program. Twenty-nine respondents contacted the psychotherapy clinic of the University of Jyväskylä via telephone or E-mail."

4a-i) Computer / Internet literacy

Computer / Internet literacy is often an implicit “de facto” eligibility criterion - this should be explicitly clarified.

	1	2	3	4	5	
subitem not at all important	○	○	○	○	○	essential

Does your paper address subitem 4a-i?

Copy and paste relevant sections from the manuscript (include quotes in quotation marks "like this" to indicate direct quotes from your manuscript), or elaborate on this item by providing additional information not in the ms, or briefly explain why the item is not applicable/relevant for your study

"Participants were recruited through an advertisement in a local newspaper seeking males aged 25- to 45-years-old with exhaustion, stress symptoms or sleeping problems. Other inclusion criteria were full time employment, basic computer skills and access to Internet. Exclusion criteria included diabetes and simultaneous attendance in another stress management program. Twenty-nine respondents contacted the psychotherapy clinic of the University of Jyväskylä via telephone or E-mail."

4a-ii) Open vs. closed, web-based vs. face-to-face assessments:

Open vs. closed, web-based vs. face-to-face assessments: Mention how participants were

recruited (online vs. offline), e.g., from an open access website or from a clinic, and clarify if this was a purely web-based trial, or there were face-to-face components (as part of the intervention or for assessment), i.e., to what degree got the study team to know the participant. In online-only trials, clarify if participants were quasi-anonymous and whether having multiple identities was possible or whether technical or logistical measures (e.g., cookies, email confirmation, phone calls) were used to detect/prevent these.

	1	2	3	4	5	
subitem not at all important	○	○	○	○	○	essential

Does your paper address subitem 4a-ii? *

Copy and paste relevant sections from the manuscript (include quotes in quotation marks "like this" to indicate direct quotes from your manuscript), or elaborate on this item by providing additional information not in the ms, or briefly explain why the item is not applicable/relevant for your study

"Participants were recruited through an advertisement in a local newspaper seeking males aged 25- to 45-years-old with exhaustion, stress symptoms or sleeping problems. Other inclusion criteria were full time employment, basic computer skills and access to Internet. Exclusion criteria included diabetes and simultaneous attendance in another stress management program. Twenty-nine respondents contacted the psychotherapy clinic of the University of Jyväskylä via telephone or E-mail." In addition to personal health technologies, such as web portal, mobile phone applications, personal monitoring devices and analysis software, participants in the intervention group received three psychologist-assisted group meetings. Participants placed in the control group did not receive any technical tools or group meetings during the study period. Pre-measurement consisting of questionnaires and heart rate variability recording was done for the intervention group before the first workshop; the control group was also measured at the same time. Both groups had an individual assessment meeting during the pre-measurement phase where they received questionnaires and were given a wearable beat-to-beat heart rate (HR) recording device (Suunto Memory Belt, Suunto Ltd., Vantaa, Finland) with instructions to do a 3-day HR variability (HRV) recording. The intervention group received feedback of the HRV recording (one-hour individual discussion of topics concerning stress, sleep and relaxation, and exercise habits) after the first workshop. Feedback of the recordings for the control group was given after the post-measurement."

4a-iii) Information giving during recruitment

Information given during recruitment. Specify how participants were briefed for recruitment and in the informed consent procedures (e.g., publish the informed consent documentation as appendix, see also item X26), as this information may have an effect on user self-selection, user expectation and may also bias results.

	1	2	3	4	5	
subitem not at all important	○	○	○	○	○	essential

Does your paper address subitem 4a-iii?

Copy and paste relevant sections from the manuscript (include quotes in quotation marks "like this" to indicate direct quotes from your manuscript), or elaborate on this item by providing additional information not in the ms, or briefly explain why the item is not applicable/relevant for your study

For recruitement, the advertisement stated that the Dept of Psychology at the University of Jyväskylä was seeking males aged 25- to 45-years-old with exhaustion, stress symptoms or sleeping problems. Other inclusion criteria were full time employment, basic computer skills and access to Internet. Exclusion criteria included diabetes and simultaneous attendance in another stress management program. The informed consent stated very clearly the procedures for the participants (the form is in Finnish, so we don't enclose it)

4b) Settings and locations where the data were collected

Does your paper address CONSORT subitem 4b? *

Copy and paste relevant sections from the manuscript (include quotes in quotation marks "like this" to indicate direct quotes from your manuscript), or elaborate on this item by providing additional information not in the ms, or briefly explain why the item is not applicable/relevant for your study

Outcomes were self-assessed through questionnaires in paper format (NOT online questionnaires).

4b-i) Report if outcomes were (self-)assessed through online questionnaires

Clearly report if outcomes were (self-)assessed through online questionnaires (as common in web-based trials) or otherwise.

	1	2	3	4	5	
subitem not at all important	○	○	○	○	○	essential

Does your paper address subitem 4b-i? *

Copy and paste relevant sections from the manuscript (include quotes in quotation marks "like this" to indicate direct quotes from your manuscript), or elaborate on this item by providing additional information not in the ms, or briefly explain why the item is not applicable/relevant for your study

"Pre-measurement consisting of questionnaires in paper format and heart rate variability recording was done for the intervention group before the first workshop; the control group was also measured at the same time."

"Two weeks after the intervention group finished its third and last workshop (e.g., after 3 months), both groups were measured for the second time (post-measurement). Follow-up questionnaires were sent to the intervention group 6 months after the intervention ended (Intervention group follow-up, n=11)."

4b-ii) Report how institutional affiliations are displayed

Report how institutional affiliations are displayed to potential participants [on ehealth media], as affiliations with prestigious hospitals or universities may affect volunteer rates, use, and reactions with regards to an intervention.(Not a required item – describe only if this may bias results)

	1	2	3	4	5	
subitem not at all important	○	○	○	○	○	essential

Does your paper address subitem 4b-ii?

Copy and paste relevant sections from the manuscript (include quotes in quotation marks "like this" to indicate direct quotes from your manuscript), or elaborate on this item by providing additional information not in the ms, or briefly explain why the item is not applicable/relevant for your study

The advertisement stated that the psychology department at the university of Jyväskylä was seeking participants.

5) The interventions for each group with sufficient details to allow replication, including how and when they were actually administered

5-i) Mention names, credential, affiliations of the developers, sponsors, and owners

Mention names, credential, affiliations of the developers, sponsors, and owners [6] (if authors/evaluators are owners or developer of the software, this needs to be declared in a “Conflict of interest” section or mentioned elsewhere in the manuscript).

	1	2	3	4	5	
subitem not at all important	○	○	○	○	○	essential

Does your paper address subitem 5-i?

Copy and paste relevant sections from the manuscript (include quotes in quotation marks "like this" to indicate direct quotes from your manuscript), or elaborate on this item by providing additional information not in the ms, or briefly explain why the item is not applicable/relevant for your study

The intervention consisted of the following technology:
Heart rate belts (Suunto Memory Belt, Suunto Ltd., Vantaa, Finland)
HRV recordings and actigraphs (Vivago Personal Wellness Manager, Vivago Ltd, Helsinki, Finland)
Mobile wellness diary, portal interface (Nokia Wellness Diary Connected Nokia Corp., Espoo, Finland)
Adaptive web-based fitness training program (Firstbeat WebTrainer, Firstbeat Technologies Ltd., Jyväskylä, Finland). Finally,
Library of evidence-based health-related information through the portal (Duodecim Health Library, Duodecim Medical Publications Ltd., Helsinki, Finland)
Mobile wellness diary (Nokia Wellness Diary, WD, Nokia, Espoo, Finland) Mobile phone version of the fitness training program (Firstbeat Mobile Coach, Firstbeat Technologies Ltd, Jyväskylä, Finland),
Mobile phone relaxation assistant which included personalized relaxation programs (SelfRelax, Relaxline, France)
Heart rate monitor (Suunto Ltd., Vantaa, Finland)
Pedometer (Omron, Kyoto, Japan)
Web platform was developed by Mawell (Oulu)
The webportal was developed by VTT Technical Research Centre of Finland
The intervention included also psychological intervention in group including commonly used CBT and ACT-methods. These are described in the Methods section. The time used for the group meetings and the time between the group meetings is described in the Methods section.

5-ii) Describe the history/development process

Describe the history/development process of the application and previous formative evaluations (e.g., focus groups, usability testing), as these will have an impact on adoption/use rates and help with interpreting results.

	1	2	3	4	5	
subitem not at all important	○	○	○	○	○	essential

Does your paper address subitem 5-ii?

Copy and paste relevant sections from the manuscript (include quotes in quotation marks "like this" to indicate direct quotes from your manuscript), or elaborate on this item by providing additional information not in the ms, or briefly explain why the item is not applicable/relevant for

your study

5-iii) Revisions and updating

Revisions and updating. Clearly mention the date and/or version number of the application/intervention (and comparator, if applicable) evaluated, or describe whether the intervention underwent major changes during the evaluation process, or whether the development and/or content was “frozen” during the trial. Describe dynamic components such as news feeds or changing content which may have an impact on the replicability of the intervention (for unexpected events see item 3b).

	1	2	3	4	5	
subitem not at all important	○	○	○	○	○	essential

Does your paper address subitem 5-iii?

Copy and paste relevant sections from the manuscript (include quotes in quotation marks "like this" to indicate direct quotes from your manuscript), or elaborate on this item by providing additional information not in the ms, or briefly explain why the item is not applicable/relevant for your study

5-iv) Quality assurance methods

Provide information on quality assurance methods to ensure accuracy and quality of information provided [1], if applicable.

	1	2	3	4	5	
subitem not at all important	○	○	○	○	○	essential

Does your paper address subitem 5-iv?

Copy and paste relevant sections from the manuscript (include quotes in quotation marks "like this" to indicate direct quotes from your manuscript), or elaborate on this item by providing additional information not in the ms, or briefly explain why the item is not applicable/relevant for your study

5-v) Ensure replicability by publishing the source code, and/or providing screenshots/screen-capture video, and/or providing flowcharts of the algorithms used

Ensure replicability by publishing the source code, and/or providing screenshots/screen-capture video, and/or providing flowcharts of the algorithms used. Replicability (i.e., other researchers should in principle be able to replicate the study) is a hallmark of scientific reporting.

	1	2	3	4	5	
subitem not at all important	○	○	○	○	○	essential

Does your paper address subitem 5-v?

Copy and paste relevant sections from the manuscript (include quotes in quotation marks "like this" to indicate direct quotes from your manuscript), or elaborate on this item by providing additional information not in the ms, or briefly explain why the item is not applicable/relevant for your study

5-vi) Digital preservation

Digital preservation: Provide the URL of the application, but as the intervention is likely to change or disappear over the course of the years; also make sure the intervention is archived (Internet Archive, webcitation.org, and/or publishing the source code or screenshots/videos alongside the article). As pages behind login screens cannot be archived, consider creating demo pages which are accessible without login.

	1	2	3	4	5	
subitem not at all important	○	○	○	○	○	essential

Does your paper address subitem 5-vi?

Copy and paste relevant sections from the manuscript (include quotes in quotation marks "like this" to indicate direct quotes from your manuscript), or elaborate on this item by providing additional information not in the ms, or briefly explain why the item is not applicable/relevant for your study

http://p4well.vtt.fi/portal (the web portal was part of the technology tools)

5-vii) Access

Access: Describe how participants accessed the application, in what setting/context, if they had to pay (or were paid) or not, whether they had to be a member of specific group. If known, describe how participants obtained "access to the platform and Internet" [1]. To ensure access for editors/reviewers/readers, consider to provide a "backdoor" login account or demo mode for reviewers/readers to explore the application (also important for archiving purposes, see vi).

	1	2	3	4	5	
subitem not at all important	O	O	O	O	O	essential

Does your paper address subitem 5-vii? *

Copy and paste relevant sections from the manuscript (include quotes in quotation marks "like this" to indicate direct quotes from your manuscript), or elaborate on this item by providing additional information not in the ms, or briefly explain why the item is not applicable/relevant for your study

"Participants were provided with the following technology tools: credentials to the web portal, mobile phones (Nokia E51) with preinstalled mobile applications, pedometers, heart rate monitors, and actigraphs." Participants were supposed to work at home, and they did'nt have to pay and they did not get paid.

5-viii) Mode of delivery, features/functionalities/components of the intervention and comparator, and the theoretical framework

Describe mode of delivery, features/functionalities/components of the intervention and comparator, and the theoretical framework [6] used to design them (instructional strategy [1], behaviour change techniques, persuasive features, etc., see e.g., [7, 8] for terminology). This includes an in-depth description of the content (including where it is coming from and who developed it) [1]," whether [and how] it is tailored to individual circumstances and allows users to track their progress and receive feedback" [6]. This also includes a description of communication delivery channels and – if computer-mediated communication is a component – whether communication was synchronous or asynchronous [6]. It also includes information on presentation strategies [1], including page design principles, average amount of text on pages, presence of hyperlinks to other resources, etc. [1].

	1	2	3	4	5	
subitem not at all important	O	O	O	O	O	essential

Does your paper address subitem 5-viii? *

Copy and paste relevant sections from the manuscript (include quotes in quotation marks "like this" to indicate direct quotes from your manuscript), or elaborate on this item by providing

additional information not in the ms, or briefly explain why the item is not applicable/relevant for your study

"The web portal provided the participants access to information, exercises, self-assessment and self-reflection tools, web-based wellness services, peer support, and expert consultation. The content of the portal was divided into modules focusing on different areas of wellbeing: Sleep, Exercise, Mood, Stress and Recovery, and Good Life. The modules consisted of five phases: information, evaluation of personal status, planning of lifestyle changes, putting the plans into action, and follow-up. In addition, the portal included a discussion forum and a messaging client for expert consultation." The portal was only a part of the technology tool kit.
Theoretical framework and behavior change techniques: "The main CBT- and ACT-based methods used in the intervention included values clarification, goal setting, self-monitoring, relaxation, mindfulness and acceptance procedures. Furthermore, regular physical activity was encouraged and emphasized as means for stress reduction, mood elevation and improved wellbeing."
"The purpose of the three mobile phone applications was to better integrate wellness management and self-monitoring into the participants' daily lives. The first application was a mobile wellness diary (Nokia Wellness Diary, WD, Nokia, Espoo, Finland) which could be used to make daily self-observations on wellness related parameters. The second application was a mobile phone version of the fitness training program (Firstbeat Mobile Coach, Firstbeat Technologies Ltd, Jyväskylä, Finland), and the third one was a mobile phone relaxation assistant which included personalized relaxation programs (SelfRelax, Relaxline, France).
The participants were encouraged to monitor their physical activity with a heart rate monitor (Suunto Ltd., Vantaa, Finland) or a pedometer (Omron, Kyoto, Japan). Heart rate monitors were primarily meant for participants who were interested in fitness training, whereas pedometers were meant to be used to measure and encourage everyday activity. Participants could enter step counts and other exercise parameters manually as daily self-observations into WD.

" The portal included a discussion forum and a messaging client for expert consultation", so the communication was partly synchronous and partly asynchronous. The participants were also contacted using email messages.

5-ix) Describe use parameters

Describe use parameters (e.g., intended “doses” and optimal timing for use). Clarify what instructions or recommendations were given to the user, e.g., regarding timing, frequency, heaviness of use, if any, or was the intervention used ad libitum.

	1	2	3	4	5	
subitem not at all important	○	○	○	○	○	essential

Does your paper address subitem 5-ix?

Copy and paste relevant sections from the manuscript (include quotes in quotation marks "like this" to indicate direct quotes from your manuscript), or elaborate on this item by providing additional information not in the ms, or briefly explain why the item is not applicable/relevant for your study

The use of parametres was one of the outcome measures.

"The personal health technologies that were provided to the participants formed a wellness toolkit from which the participants could choose the most appropriate ones for their needs and preferences."
The participants had the chance to use the technology tools as much as they wanted, whenever they wanted, and they could choose the most appropriate ones.

5-x) Clarify the level of human involvement

Clarify the level of human involvement (care providers or health professionals, also technical assistance) in the e-intervention or as co-intervention (detail number and expertise of professionals involved, if any, as well as “type of assistance offered, the timing and frequency of the support, how it is initiated, and the medium by which the assistance is delivered”. It may be necessary to distinguish between the level of human involvement required for the trial, and the level of human involvement required for a routine application outside of a RCT setting (discuss under item 21 – generalizability).

	1	2	3	4	5	
subitem not at all important	○	○	○	○	○	essential

Does your paper address subitem 5-x?

Copy and paste relevant sections from the manuscript (include quotes in quotation marks "like this" to indicate direct quotes from your manuscript), or elaborate on this item by providing additional information not in the ms, or briefly explain why the item is not applicable/relevant for your study

"In addition to personal health technologies, such as web portal, mobile phone applications, personal monitoring devices and analysis software, participants in the intervention group received three psychologist-assisted group meetings. Participants placed in the control group did not receive any technical tools or group meetings during the study period. Pre-measurement consisting of questionnaires in paper format and heart rate variability recording was done for the intervention group before the first workshop; the control group was also measured at the same time. Both groups had an individual assessment meeting during the pre-measurement phase where they received questionnaires and were given a wearable beat-to-beat heart rate (HR) recording device (Suunto Memory Belt, Suunto Ltd., Vantaa, Finland) with instructions to do a 3-day HR variability (HRV) recording. One week after the assessment meeting the questionnaires and the heart rate belts were collected and analyzed. The intervention group received feedback of the HRV recording (one-hour individual discussion of topics concerning stress, sleep and relaxation, and exercise habits) after the first workshop. Feedback of the recordings for the control group was given after the post-measurement. The feedback was given by an exercise physiologist. "
Technical assistance was given by the VTT Technical Research Centre, when needed.

5-xi) Report any prompts/reminders used

Report any prompts/reminders used: Clarify if there were prompts (letters, emails, phone calls, SMS) to use the application, what triggered them, frequency etc. It may be necessary to distinguish between the level of prompts/reminders required for the trial, and the level of prompts/reminders for a routine application outside of a RCT setting (discuss under item 21 – generalizability).

	1	2	3	4	5	
subitem not at all important	O	O	O	O	O	essential

Does your paper address subitem 5-xi? *

Copy and paste relevant sections from the manuscript (include quotes in quotation marks "like this" to indicate direct quotes from your manuscript), or elaborate on this item by providing additional information not in the ms, or briefly explain why the item is not applicable/relevant for your study

No reminders or promts were used

5-xii) Describe any co-interventions (incl. training/support)

Describe any co-interventions (incl. training/support): Clearly state any interventions that are provided in addition to the targeted eHealth intervention, as ehealth intervention may not be designed as stand-alone intervention. This includes training sessions and support [1]. It may be necessary to distinguish between the level of training required for the trial, and the level of training for a routine application outside of a RCT setting (discuss under item 21 – generalizability.

	1	2	3	4	5	
subitem not at all important	O	O	O	O	O	essential

Does your paper address subitem 5-xii? *

Copy and paste relevant sections from the manuscript (include quotes in quotation marks "like this" to indicate direct quotes from your manuscript), or elaborate on this item by providing additional information not in the ms, or briefly explain why the item is not applicable/relevant for your study

No co-interventions were provided

6a) Completely defined pre-specified primary and secondary outcome measures, including how and when they were assessed

Does your paper address CONSORT subitem 6a? *

Copy and paste relevant sections from the manuscript (include quotes in quotation marks "like this" to indicate direct quotes from your manuscript), or elaborate on this item by providing additional information not in the ms, or briefly explain why the item is not applicable/relevant for your study

No online questionnaires were used. Questionnaires used were internationally known and validated questionnaires

6a-i) Online questionnaires: describe if they were validated for online use and apply CHERRIES items to describe how the questionnaires were designed/deployed

If outcomes were obtained through online questionnaires, describe if they were validated for online use and apply CHERRIES items to describe how the questionnaires were designed/deployed [9].

	1	2	3	4	5	
subitem not at all important	○	○	○	○	○	essential

Does your paper address subitem 6a-i?

Copy and paste relevant sections from manuscript text

Questionnaires used were internationally known and validated questionnaires. No online questionnaires were used.
"Pre-measurement consisting of self-assessed questionnaires in paper format and heart rate variability recording was done for the intervention group before the first workshop; the control group was also measured at the same time."
"Two weeks after the intervention group finished its third and last workshop (e.g., after 3 months), both groups were measured for the second time (post-measurement). Follow-up questionnaires were sent to the intervention group 6 months after the intervention ended (Intervention group follow-up, n=11)."

6a-ii) Describe whether and how “use” (including intensity of use/dosage) was defined/measured/monitored

Describe whether and how “use” (including intensity of use/dosage) was defined/measured/monitored (logins, logfile analysis, etc.). Use/adoption metrics are important process outcomes that should be reported in any ehealth trial.

	1	2	3	4	5	
subitem not at all important	○	○	○	○	○	essential

Does your paper address subitem 6a-ii?

Copy and paste relevant sections from manuscript text

"Usage logs were collected from the portal and the mobile applications after the end of the intervention. For pedometers and heart rate monitors, usage frequency was assessed through the post-intervention questionnaire. Participants were defined as active users of a given tool if they had used it during at least half of the study weeks (based on log data) or reported having used it at least weekly (questionnaire data)."

6a-iii) Describe whether, how, and when qualitative feedback from participants was obtained

Describe whether, how, and when qualitative feedback from participants was obtained (e.g., through emails, feedback forms, interviews, focus groups).

	1	2	3	4	5	
subitem not at all important	○	○	○	○	○	essential

Does your paper address subitem 6a-iii?

Copy and paste relevant sections from manuscript text

"User experiences were measured post-intervention with a questionnaire about perceived utility and acceptance of each individual technology tool, and the perceived usefulness of different intervention components. The perceived utility of the intervention as a whole was assessed with questions about perceived benefits from participation in the study."

6b) Any changes to trial outcomes after the trial commenced, with reasons

Does your paper address CONSORT subitem 6b? *

Copy and paste relevant sections from the manuscript (include quotes in quotation marks "like this" to indicate direct quotes from your manuscript), or elaborate on this item by providing additional information not in the ms, or briefly explain why the item is not applicable/relevant for your study

No changes

7a) How sample size was determined

NPT: When applicable, details of whether and how the clustering by care provides or centers was addressed

7a-i) Describe whether and how expected attrition was taken into account when calculating the sample size

Describe whether and how expected attrition was taken into account when calculating the sample size.

	1	2	3	4	5	
subitem not at all important	○	○	○	○	○	essential

Does your paper address subitem 7a-i?

Copy and paste relevant sections from manuscript title (include quotes in quotation marks "like this" to indicate direct quotes from your manuscript), or elaborate on this item by providing additional information not in the ms, or briefly explain why the item is not applicable/relevant for your study

"Of the 25 male participants, we excluded one participant from analysis because of not fulfilling the inclusion criteria (because of age and retirement) and one participant who did not participate in the follow-up measurements. One participant in the intervention group was lost at follow-up (see flow diagram). Thus, the total number of participants included in the study was 23. Dropout (n=1) was included in the results and treated with an intention-to-treat-analysis using the data missing principle of last observation carried forward."

7b) When applicable, explanation of any interim analyses and stopping guidelines

Does your paper address CONSORT subitem 7b? *

Copy and paste relevant sections from the manuscript (include quotes in quotation marks "like this" to indicate direct quotes from your manuscript), or elaborate on this item by providing additional information not in the ms, or briefly explain why the item is not applicable/relevant for your study

8a) Method used to generate the random allocation sequence

NPT: When applicable, how care providers were allocated to each trial group

Does your paper address CONSORT subitem 8a? *

Copy and paste relevant sections from the manuscript (include quotes in quotation marks "like this" to indicate direct quotes from your manuscript), or elaborate on this item by providing additional information not in the ms, or briefly explain why the item is not applicable/relevant for your study

Randomization

8b) Type of randomisation; details of any restriction (such as blocking and block size)

Does your paper address CONSORT subitem 8b? *

Copy and paste relevant sections from the manuscript (include quotes in quotation marks "like this" to indicate direct quotes from your manuscript), or elaborate on this item by providing additional information not in the ms, or briefly explain why the item is not applicable/relevant for your study

"We then randomly allocated participants either to the intervention or the waiting-list control group (intervention group=11, control group=12) as follows: First, the sample was divided into pairs according to the depression score, the participants within each pair having about the same score (as measured by Beck's Depression Inventory BDI) [38]. Second, the order of pairs was randomized. Third, participants within pairs were randomly assigned either to intervention or control group. Thus, the groups were made equal on the basis of reported depressive symptoms."

9) Mechanism used to implement the random allocation sequence (such as sequentially numbered containers), describing any steps taken to conceal the sequence until interventions were assigned

Does your paper address CONSORT subitem 9? *

Copy and paste relevant sections from the manuscript (include quotes in quotation marks "like this" to indicate direct quotes from your manuscript), or elaborate on this item by providing additional information not in the ms, or briefly explain why the item is not applicable/relevant for your study

"We then randomly allocated participants either to the intervention or the waiting-list control group (intervention group=11, control group=12) as follows: First, the sample was divided into pairs according to the depression score, the participants within each pair having about the same score (as measured by Beck's Depression Inventory BDI) [38]. Second, the order of pairs was randomized. Third, participants within pairs were randomly assigned either to intervention or control group. Thus, the groups were made equal on the basis of reported depressive symptoms."

10) Who generated the random allocation sequence, who enrolled participants, and who assigned participants to interventions

Does your paper address CONSORT subitem 10? *

Copy and paste relevant sections from the manuscript (include quotes in quotation marks "like this" to indicate direct quotes from your manuscript), or elaborate on this item by providing additional information not in the ms, or briefly explain why the item is not applicable/relevant for your study

"The randomization was generated by the researchers."

11a) If done, who was blinded after assignment to interventions (for example, participants, care providers, those assessing outcomes) and how

NPT: Whether or not administering co-interventions were blinded to group assignment

11a-i) Specify who was blinded, and who wasn't

Specify who was blinded, and who wasn't. Usually, in web-based trials it is not possible to blind the participants [1, 3] (this should be clearly acknowledged), but it may be possible to blind outcome assessors, those doing data analysis or those administering co-interventions (if any).

	1	2	3	4	5	
subitem not at all important	○	○	○	○	○	essential

Does your paper address subitem 11a-i? *

Copy and paste relevant sections from the manuscript (include quotes in quotation marks "like this" to indicate direct quotes from your manuscript), or elaborate on this item by providing additional information not in the ms, or briefly explain why the item is not applicable/relevant for your study

In this personal health technology intervention it was not possible to blind the partipants. We were not able to use blind outcome assessors.

11a-ii) Discuss e.g., whether participants knew which intervention was the "intervention of interest" and which one was the "comparator"

Informed consent procedures (4a-ii) can create biases and certain expectations - discuss e.g., whether participants knew which intervention was the "intervention of interest" and which one was the "comparator".

	1	2	3	4	5	
subitem not at all important	○	○	○	○	○	essential

Does your paper address subitem 11a-ii?

Copy and paste relevant sections from the manuscript (include quotes in quotation marks "like this" to indicate direct quotes from your manuscript), or elaborate on this item by providing additional information not in the ms, or briefly explain why the item is not applicable/relevant for your study

Participants were informed after allocation in which group, study group or control group, they were allocated to.

11b) If relevant, description of the similarity of interventions

(this item is usually not relevant for ehealth trials as it refers to similarity of a placebo or sham intervention to a active medication/intervention)

Does your paper address CONSORT subitem 11b? *

Copy and paste relevant sections from the manuscript (include quotes in quotation marks "like this" to indicate direct quotes from your manuscript), or elaborate on this item by providing additional information not in the ms, or briefly explain why the item is not applicable/relevant for your study

not relevant

12a) Statistical methods used to compare groups for primary and secondary outcomes

NPT: When applicable, details of whether and how the clustering by care providers or centers was addressed

Does your paper address CONSORT subitem 12a? *

Copy and paste relevant sections from the manuscript (include quotes in quotation marks "like this" to indicate direct quotes from your manuscript), or elaborate on this item by providing additional information not in the ms, or briefly explain why the item is not applicable/relevant for your study

Dropout (n=1) was treated with an intention-to-treat-analysis using the data missing principle of last observation carried forward."

12a-i) Imputation techniques to deal with attrition / missing values

Imputation techniques to deal with attrition / missing values: Not all participants will use the intervention/comparator as intended and attrition is typically high in ehealth trials. Specify how participants who did not use the application or dropped out from the trial were treated in the statistical analysis (a complete case analysis is strongly discouraged, and simple imputation techniques such as LOCF may also be problematic [4]).

	1	2	3	4	5	
subitem not at all important	○	○	○	○	○	essential

Does your paper address subitem 12a-i? *

Copy and paste relevant sections from the manuscript (include quotes in quotation marks "like this" to indicate direct quotes from your manuscript), or elaborate on this item by providing additional information not in the ms, or briefly explain why the item is not applicable/relevant for your study

Dropout (n=1) was treated with an intention-to-treat-analysis using the data missing principle of last observation carried forward."

12b) Methods for additional analyses, such as subgroup analyses and adjusted analyses

Does your paper address CONSORT subitem 12b? *

Copy and paste relevant sections from the manuscript (include quotes in quotation marks "like this" to indicate direct quotes from your manuscript), or elaborate on this item by providing additional information not in the ms, or briefly explain why the item is not applicable/relevant for your study

Not relevant because of the small sample size.

X26) REB/IRB Approval and Ethical Considerations [recommended as subheading under "Methods"] (not a CONSORT item)

X26-i) Comment on ethics committee approval

	1	2	3	4	5	
subitem not at all important	○	○	○	○	○	essential

Does your paper address subitem X26-i?

Copy and paste relevant sections from the manuscript (include quotes in quotation marks "like this" to indicate direct quotes from your manuscript), or elaborate on this item by providing additional information not in the ms, or briefly explain why the item is not applicable/relevant for your study

"The Research Ethics Committee of the University of Jyväskylä approved this study."
We did not have any problems with our application to the Research Ethics Committee of the University of Jyväskylä.

x26-ii) Outline informed consent procedures

Outline informed consent procedures e.g., if consent was obtained offline or online (how? Checkbox, etc.?), and what information was provided (see 4a-ii). See [6] for some items to be included in informed consent documents.

	1	2	3	4	5	
subitem not at all important	○	○	○	○	○	essential

Does your paper address subitem X26-ii?

Copy and paste relevant sections from the manuscript (include quotes in quotation marks "like this" to indicate direct quotes from your manuscript), or elaborate on this item by providing additional information not in the ms, or briefly explain why the item is not applicable/relevant for your study

"Consent from participants was obtained offline in paper format. Participants received detailed information about the study procedure and their rights."

X26-iii) Safety and security procedures

Safety and security procedures, incl. privacy considerations, and any steps taken to reduce the likelihood or detection of harm (e.g., education and training, availability of a hotline)

	1	2	3	4	5	
subitem not at all important	○	○	○	○	○	essential

Does your paper address subitem X26-iii?

Copy and paste relevant sections from the manuscript (include quotes in quotation marks "like this" to indicate direct quotes from your manuscript), or elaborate on this item by providing additional information not in the ms, or briefly explain why the item is not applicable/relevant for your study

The web portal including personal information was secured: "The secured web portal provided the participants access to information, exercises, self-assessment and self-reflection tools, web-based wellness services, peer support, and expert consultation."

RESULTS

13a) For each group, the numbers of participants who were randomly assigned, received intended treatment, and were analysed for the primary outcome

NPT: The number of care providers or centers performing the intervention in each group and the number of patients treated by each care provider in each center

Does your paper address CONSORT subitem 13a? *

Copy and paste relevant sections from the manuscript (include quotes in quotation marks "like this" to indicate direct quotes from your manuscript), or elaborate on this item by providing additional information not in the ms, or briefly explain why the item is not applicable/relevant for your study

Thus, the total number of participants included in the study was 23. Dropout (n=1) was treated with an intention-to-treat-analysis using the data missing principle of last observation carried forward.

13b) For each group, losses and exclusions after randomisation, together with reasons

Does your paper address CONSORT subitem 13b? (NOTE: Preferably, this is shown in a CONSORT flow diagram) *

Copy and paste relevant sections from the manuscript (include quotes in quotation marks "like this" to indicate direct quotes from your manuscript), or elaborate on this item by providing additional information not in the ms, or briefly explain why the item is not applicable/relevant for your study

One participant in the intervention group was lost at follow-up (he did not give any reason). Thus, the total number of participants included in the study was 23 (see flow diagram)

13b-i) Attrition diagram

Strongly recommended: An attrition diagram (e.g., proportion of participants still logging in or using the intervention/comparator in each group plotted over time, similar to a survival curve) or other figures or tables demonstrating usage/dose/engagement.

	1	2	3	4	5	
subitem not at all important	○	○	○	○	○	essential

Does your paper address subitem 13b-i?

Copy and paste relevant sections from the manuscript or cite the figure number if applicable (include quotes in quotation marks "like this" to indicate direct quotes from your manuscript), or elaborate on this item by providing additional information not in the ms, or briefly explain why the item is not applicable/relevant for your study

One participant in the intervention group was lost at follow-up (he did not give any reason). Thus, the total number of participants included in the study was 23 (see flow diagram)

14a) Dates defining the periods of recruitment and follow-up

Does your paper address CONSORT subitem 14a? *

Copy and paste relevant sections from the manuscript (include quotes in quotation marks "like this" to indicate direct quotes from your manuscript), or elaborate on this item by providing additional information not in the ms, or briefly explain why the item is not applicable/relevant for your study

"The study took place at the psychotherapy clinic of the department of psychology at the University of Jyväskylä, Finland, from January 2009 to October 2009."

14a-i) Indicate if critical “secular events” fell into the study period

Indicate if critical “secular events” fell into the study period, e.g., significant changes in Internet resources available or “changes in computer hardware or Internet delivery resources”

	1	2	3	4	5	
subitem not at all important	○	○	○	○	○	essential

Does your paper address subitem 14a-i?

Copy and paste relevant sections from the manuscript (include quotes in quotation marks "like this" to indicate direct quotes from your manuscript), or elaborate on this item by providing additional information not in the ms, or briefly explain why the item is not applicable/relevant for

your study

No critical events during the study period.

14b) Why the trial ended or was stopped (early)

Does your paper address CONSORT subitem 14b? *

Copy and paste relevant sections from the manuscript (include quotes in quotation marks "like this" to indicate direct quotes from your manuscript), or elaborate on this item by providing additional information not in the ms, or briefly explain why the item is not applicable/relevant for your study

Not relevant

15) A table showing baseline demographic and clinical characteristics for each group

NPT: When applicable, a description of care providers (case volume, qualification, expertise, etc.) and centers (volume) in each group

Does your paper address CONSORT subitem 15? *

Copy and paste relevant sections from the manuscript (include quotes in quotation marks "like this" to indicate direct quotes from your manuscript), or elaborate on this item by providing additional information not in the ms, or briefly explain why the item is not applicable/relevant for your study

"The mean age of the participants was 47.1 years in the intervention group and 39.4 in the control group (Table 1). The intervention group was older, $t(21) = 2.78$, $P < 0.05$, and had a lower BMI, $t(21) = 2.42$, $P < 0.05$, than the control group. The groups did not differ in regard to education, type of work, shift work or reported depressive symptoms."

Additional demographic data is presented in table form.

15-i) Report demographics associated with digital divide issues

In ehealth trials it is particularly important to report demographics associated with digital divide issues, such as age, education, gender, social-economic status, computer/Internet/ehealth literacy of the participants, if known.

	1	2	3	4	5	
subitem not at all important	○	○	○	○	○	essential

Does your paper address subitem 15-i? *

Copy and paste relevant sections from the manuscript (include quotes in quotation marks "like this" to indicate direct quotes from your manuscript), or elaborate on this item by providing additional information not in the ms, or briefly explain why the item is not applicable/relevant for your study

"The mean age of the participants was 47.1 years in the intervention group and 39.4 in the control group (Table 1). The intervention group was older, $t(21) = 2.78$, $P < 0.05$, and had a lower BMI, $t(21) = 2.42$, $P < 0.05$, than the control group. The groups did not differ in regard to education, type of work, shift work or reported depressive symptoms."

16) For each group, number of participants (denominator) included in each analysis and whether the analysis was by original assigned groups

16-i) Report multiple “denominators” and provide definitions

Report multiple “denominators” and provide definitions: Report N’s (and effect sizes) “across a range of study participation [and use] thresholds” [1], e.g., N exposed, N consented, N used more than x times, N used more than y weeks, N participants “used” the intervention/comparator at specific pre-defined time points of interest (in absolute and relative numbers per group). Always clearly define “use” of the intervention.

	1	2	3	4	5	
subitem not at all important	○	○	○	○	○	essential

Does your paper address subitem 16-i? *

Copy and paste relevant sections from the manuscript (include quotes in quotation marks "like this" to indicate direct quotes from your manuscript), or elaborate on this item by providing additional information not in the ms, or briefly explain why the item is not applicable/relevant for your study

The number of participants defined as active users are reported:

"The most common benefits the participants reported included increased willingness to improve personal wellbeing (8/11), decreased level of stress (6/11), and increased amount of exercise (5/11). The most useful intervention components were considered to be measurements and feedback (10/11), personal monitoring devices (9/11), group meetings (8/11), and mobile applications (6/11)."
"All participants tried at least three out of 6 available tools (mean = 4.7, range 3-6) and 10/11 participants used at least one of the tools (mean = 1.9, range 1-4) actively. Each tool had at least one active user (Table 2). The mobile relaxation application had the highest number of active users. Pedometer was ranked as the easiest and most personally suitable. Heart rate monitor was rated as the most useful and difficult to use."
Mean values of outcome measurements and statistical analysis, as well as effect sizes are presented in Table 3 and 4.

16-ii) Primary analysis should be intent-to-treat

Primary analysis should be intent-to-treat, secondary analyses could include comparing only “users”, with the appropriate caveats that this is no longer a randomized sample (see 18-i).

	1	2	3	4	5	
subitem not at all important	○	○	○	○	○	essential

Does your paper address subitem 16-ii?

Copy and paste relevant sections from the manuscript (include quotes in quotation marks "like this" to indicate direct quotes from your manuscript), or elaborate on this item by providing additional information not in the ms, or briefly explain why the item is not applicable/relevant for your study

Intent-to-treat analyses was used as a primary analyses. "One participant in the intervention group was lost at follow-up (see flow diagram). Thus, the total number of participants included in the study was 23. Dropout (n=1) was treated with an intention-to-treat-analyses using the data missing principle of last observation carried forward."

17a) For each primary and secondary outcome, results for each group, and the estimated effect size and its precision (such as 95% confidence interval)

Does your paper address CONSORT subitem 17a? *

Copy and paste relevant sections from the manuscript (include quotes in quotation marks "like this" to indicate direct quotes from your manuscript), or elaborate on this item by providing additional information not in the ms, or briefly explain why the item is not applicable/relevant for your study

In addition to mean values and standard deviations, 95 % confidence intervals and effect sizes are presented in Table 3 and 4.

17a-i) Presentation of process outcomes such as metrics of use and intensity of use

In addition to primary/secondary (clinical) outcomes, the presentation of process outcomes such as metrics of use and intensity of use (dose, exposure) and their operational definitions is critical. This does not only refer to metrics of attrition (13-b) (often a binary variable), but also to more continuous exposure metrics such as “average session length”. These must be accompanied by a technical description how a metric like a “session” is defined (e.g., timeout after idle time) [1] (report under item 6a).

	1	2	3	4	5	
subitem not at all important	○	○	○	○	○	essential

Does your paper address subitem 17a-i?

Copy and paste relevant sections from the manuscript (include quotes in quotation marks "like this" to indicate direct quotes from your manuscript), or elaborate on this item by providing additional information not in the ms, or briefly explain why the item is not applicable/relevant for your study

In addition to mean values and standard deviations, 95 % confidence intervals and effect sizes are presented in Table 3 and 4.

17b) For binary outcomes, presentation of both absolute and relative effect sizes is recommended

Does your paper address CONSORT subitem 17b? *

Copy and paste relevant sections from the manuscript (include quotes in quotation marks "like this" to indicate direct quotes from your manuscript), or elaborate on this item by providing additional information not in the ms, or briefly explain why the item is not applicable/relevant for your study

Not relevant

18) Results of any other analyses performed, including subgroup analyses and adjusted analyses, distinguishing pre-specified from exploratory

Does your paper address CONSORT subitem 18? *

Copy and paste relevant sections from the manuscript (include quotes in quotation marks "like this" to indicate direct quotes from your manuscript), or elaborate on this item by providing additional information not in the ms, or briefly explain why the item is not applicable/relevant for your study

Not relevant because of small sample size.

18-i) Subgroup analysis of comparing only users

A subgroup analysis of comparing only users is not uncommon in ehealth trials, but if done, it must be stressed that this is a self-selected sample and no longer an unbiased sample from a randomized trial (see 16-iii).

	1	2	3	4	5	
subitem not at all important	○	○	○	○	○	essential

Does your paper address subitem 18-i?

Copy and paste relevant sections from the manuscript (include quotes in quotation marks "like this" to indicate direct quotes from your manuscript), or elaborate on this item by providing additional information not in the ms, or briefly explain why the item is not applicable/relevant for your study

Not relevant

19) All important harms or unintended effects in each group

(for specific guidance see CONSORT for harms)

Does your paper address CONSORT subitem 19? *

Copy and paste relevant sections from the manuscript (include quotes in quotation marks "like this" to indicate direct quotes from your manuscript), or elaborate on this item by providing additional information not in the ms, or briefly explain why the item is not applicable/relevant for your study

No technical problems

19-i) Include privacy breaches, technical problems

Include privacy breaches, technical problems. This does not only include physical “harm” to participants, but also incidents such as perceived or real privacy breaches [1], technical problems, and other unexpected/unintended incidents. “Unintended effects” also includes unintended positive effects [2].

	1	2	3	4	5	
subitem not at all important	○	○	○	○	○	essential

Does your paper address subitem 19-i?

Copy and paste relevant sections from the manuscript (include quotes in quotation marks "like this" to indicate direct quotes from your manuscript), or elaborate on this item by providing additional information not in the ms, or briefly explain why the item is not applicable/relevant for your study

No technical problems

19-ii) Include qualitative feedback from participants or observations from staff/researchers

Include qualitative feedback from participants or observations from staff/researchers, if available, on strengths and shortcomings of the application, especially if they point to unintended/unexpected effects or uses. This includes (if available) reasons for why people did or did not use the application as intended by the developers.

	1	2	3	4	5	
subitem not at all important	○	○	○	○	○	essential

Does your paper address subitem 19-ii?

Copy and paste relevant sections from the manuscript (include quotes in quotation marks "like this" to indicate direct quotes from your manuscript), or elaborate on this item by providing additional information not in the ms, or briefly explain why the item is not applicable/relevant for your study

Qualitative feedback from participants is presented in the beginning of the Results section: "All participants in the intervention group stated that their well-being had improved as a result of the intervention. The most common benefits the participants reported included increased willingness to improve personal wellbeing (8/11), decreased level of stress (6/11), and increased amount of exercise (5/11). The most useful intervention components were considered to be measurements and feedback (10/11), personal monitoring devices (9/11), group meetings (8/11), and mobile applications (6/11).
All participants tried at least three out of 6 available tools (mean = 4.7, range 3-6) and 10/11 participants used at least one of the tools (mean = 1.9, range 1-4) actively. Each tool had at least one active user (Table 2). The mobile relaxation application had the highest number of active users. Pedometer was ranked as the easiest and most personally suitable. Heart rate monitor was rated as the most useful and difficult to use."

DISCUSSION

22) Interpretation consistent with results, balancing benefits and harms, and considering other relevant evidence

NPT: In addition, take into account the choice of the comparator, lack of or partial blinding, and unequal expertise of care providers or centers in each group

22-i) Restate study questions and summarize the answers suggested by the data, starting with primary outcomes and process outcomes (use)

Restate study questions and summarize the answers suggested by the data, starting with primary outcomes and process outcomes (use).

	1	2	3	4	5	
subitem not at all important	○	○	○	○	○	essential

Does your paper address subitem 22-i? *

Copy and paste relevant sections from the manuscript (include quotes in quotation marks "like this" to indicate direct quotes from your manuscript), or elaborate on this item by providing additional information not in the ms, or briefly explain why the item is not applicable/relevant for your study

"The objective of this study was to study the feasibility of the developed P4Well intervention among male, working-age citizens who experienced mild to moderate symptoms of stress and/or depression. We assessed the effects of the intervention using depression, psychological symptoms and stress as primary outcome measures. Secondary outcome measures included quality of life, psychological flexibility and job strain. Furthermore, we studied the acceptability and usage of the intervention and its components."
"Depressive symptoms, as measured by BDI, decreased more in the intervention group compared to the control group (Table 3). There was a marginally significant group by time interaction effect for BDI (P = 0.072). The mean BDI value decreased with more than eight scores (Confidence Interval (CI) = 4.92; 11.99) in the treatment group compared to four scores (CI = 0.62; 7.38) in the control group. We found a medium Effect size (ES) (d = 0.57) between groups in favor of the intervention group. Participants maintained positive changes at the 6-month follow-up. We found a significant within-group effect over time for the intervention group (P = 0.001): both post- and follow-up measurements were significantly lower compared to the BDI pre-measurement. Pre- to follow-up BDI measurements indicated a large within-group ES (d = 1.11). An analysis of the number of the participants who reported depressive symptoms at pre-, post- and follow-up measurements also suggested that the intervention had a positive effect on mood. Seven participants (64%) in the intervention group and six (55%) in the control group reported at least mild depression (a BDI of at least 10) at the beginning of the study. Only one person (9%) reported depressive symptoms in the intervention group after the intervention ended. In the control group, six people (55%) were still depressed at post-measurement. At follow-up, only one person in the intervention group reported BDI values greater than 10.
Psychological symptoms (SCL-90) decreased in the intervention group but remained at the same level in the control group (Table 3). We found a marginally significant group by time interaction effect in psychological symptoms (P = 0.053). The between-group ES was small (d = 0.39). The within-group ES from pre- to follow-up measurement was medium (d = 1.07). Again, we found a significant within-group effect for the intervention group: both the post- and follow-up measurements were significantly lower compared to pre-measurement scores. A significant group by time interaction effect was found for health (P = 0.008) and working ability (P = 0.016). The between- and within-group ESs were small for both health (d = 0.38 and 0.56, respectively) and working ability (d = 0.21 and 0.60, respectively). Furthermore, for these variables we found a significant within-group effect in the intervention group. Thus, health was rated higher after treatment, and participants estimated their working ability to be higher at follow-up compared to the beginning of the treatment. We did not observe a significant group by time interaction for burnout (Table 4). However, the burnout scores decreased from pre-measurement to follow-up and showed a medium ES (d = 0.91). In the intervention group we found a significant within-group effect on cynicism, although there was no significant group by time interaction. However, we obtained a medium (between group) ES for cynicism. There were marginally significant interaction effects on effort (P = 0.07) and over-commitment (P = 0.08). The scores for over-commitment were lower at follow-up compared to the beginning of treatment. The within-group ES from pre-measurement to follow-up was small for over-commitment (d = 0.61).

22-ii) Highlight unanswered new questions, suggest future research

Highlight unanswered new questions, suggest future research.

	1	2	3	4	5	
subitem not at all important	○	○	○	○	○	essential

Does your paper address subitem 22-ii?

Copy and paste relevant sections from the manuscript (include quotes in quotation marks "like this" to indicate direct quotes from your manuscript), or elaborate on this item by providing additional information not in the ms, or briefly explain why the item is not applicable/relevant for your study

"Future studies need to investigate the effectiveness, benefits and possible problems of psychological interventions which incorporate new technologies."

20) Trial limitations, addressing sources of potential bias, imprecision, and, if relevant, multiplicity of analyses

20-i) Typical limitations in ehealth trials

Typical limitations in ehealth trials: Participants in ehealth trials are rarely blinded. Ehealth trials often look at a multiplicity of outcomes, increasing risk for a Type I error. Discuss biases due to non-use of the intervention/usability issues, biases through informed consent procedures, unexpected events.

	1	2	3	4	5	
subitem not at all important	○	○	○	○	○	essential

Does your paper address subitem 20-i? *

Copy and paste relevant sections from the manuscript (include quotes in quotation marks "like this" to indicate direct quotes from your manuscript), or elaborate on this item by providing additional information not in the ms, or briefly explain why the item is not applicable/relevant for your study

"This study had several limitations. First of all, the number of participants was small and therefore the statistical power of the study is weak. This lack of power affects our ability to detect differences between the intervention and control group as well as our ability to generalize the results. Also, most participants reported a small number of psychological problems at the beginning of the study. Thus, the possibility for improvement was small (for example for AAQ, BDI and SCL-90) suggesting that other measurements may have been more appropriate for observing the changes. The control group showed also some improvement that was possibly due to assessment procedures at the beginning. The effectiveness and the acceptability of this intervention need also to be investigated in other populations reporting more severe problems. Furthermore, longer follow-up periods may be needed to ensure the sustainability of the effect. Overall, because all the participants in this study were male, our results can be generalized to only middle-aged men who seek help for stress-related problems and mild to moderate depression. Additionally, participants were provided several technology tools within a short period of time, which caused cognitive load that may have hindered participants' capability and motivation to discover personally suitable tools. Some tools also had usability problems, data entered in one application was not synchronized to others, and most of self-monitoring was done manually. Since the time the study was conducted, there have been considerable advances especially in smartphone technology which would allow a more integrated and usable technology toolkit."

21) Generalisability (external validity, applicability) of the trial findings

NPT: External validity of the trial findings according to the intervention, comparators, patients, and care providers or centers involved in the trial

21-i) Generalizability to other populations

Generalizability to other populations: In particular, discuss generalizability to a general Internet population, outside of a RCT setting, and general patient population, including applicability of the study results for other organizations

	1	2	3	4	5	
subitem not at all important	○	○	○	○	○	essential

Does your paper address subitem 21-i?

Copy and paste relevant sections from the manuscript (include quotes in quotation marks "like this" to indicate direct quotes from your manuscript), or elaborate on this item by providing additional information not in the ms, or briefly explain why the item is not applicable/relevant for your study

"Overall, because all the participants in this study were male, our results can be generalized to only middle-aged men who seek help for stress-related problems and mild to moderate depression."

21-ii) Discuss if there were elements in the RCT that would be different in a routine application setting

Discuss if there were elements in the RCT that would be different in a routine application setting (e.g., prompts/reminders, more human involvement, training sessions or other co-interventions) and what impact the omission of these elements could have on use, adoption, or outcomes if the intervention is applied outside of a RCT setting.

	1	2	3	4	5	
subitem not at all important	○	○	○	○	○	essential

Does your paper address subitem 21-ii?

Copy and paste relevant sections from the manuscript (include quotes in quotation marks "like this" to indicate direct quotes from your manuscript), or elaborate on this item by providing additional information not in the ms, or briefly explain why the item is not applicable/relevant for your study

"Our intervention may be a noteworthy tool for self-management, health-related prevention and general well-being in health care settings. Prevention and early intervention based on self-management are especially important given that healthcare resources are limited."

OTHER INFORMATION

23) Registration number and name of trial registry

Does your paper address CONSORT subitem 23? *

Copy and paste relevant sections from the manuscript (include quotes in quotation marks "like this" to indicate direct quotes from your manuscript), or elaborate on this item by providing additional information not in the ms, or briefly explain why the item is not applicable/relevant for your study

Tthe Finnish Funding Agency for Technology and Innovation (TEKES) project number 40011/08

24) Where the full trial protocol can be accessed, if available

Does your paper address CONSORT subitem 24? *

Cite a Multimedia Appendix, other reference, or copy and paste relevant sections from the manuscript (include quotes in quotation marks "like this" to indicate direct quotes from your manuscript), or elaborate on this item by providing additional information not in the ms, or briefly explain why the item is not applicable/relevant for your study

Final report of the P4Well Research Project (18 Jan 2010): Pervasive and Personal Psychophysiological Wellbeing and Recovery Management Concept based on Stress, Sleep and Exercise by VTT Technical Researc Centre of Finland.

25) Sources of funding and other support (such as supply of drugs), role of funders

Does your paper address CONSORT subitem 25? *

Copy and paste relevant sections from the manuscript (include quotes in quotation marks "like this" to indicate direct quotes from your manuscript), or elaborate on this item by providing additional information not in the ms, or briefly explain why the item is not applicable/relevant for your study

The study was funded by the Finnish Funding Agency for Technology and Innovation (TEKES).

X27) Conflicts of Interest (not a CONSORT item)

X27-i) State the relation of the study team towards the system being evaluated

In addition to the usual declaration of interests (financial or otherwise), also state the relation of the study team towards the system being evaluated, i.e., state if the authors/evaluators are distinct from or identical with the developers/sponsors of the intervention.

	1	2	3	4	5	
subitem not at all important	○	○	○	○	○	essential

Does your paper address subitem X27-i?

Copy and paste relevant sections from the manuscript (include quotes in quotation marks "like this" to indicate direct quotes from your manuscript), or elaborate on this item by providing additional information not in the ms, or briefly explain why the item is not applicable/relevant for your study

No competing interests

About the CONSORT EHEALTH checklist

As a result of using this checklist, did you make changes in your manuscript? *

- ○ yes, major changes
- ○ yes, minor changes
- ○ no

What were the most important changes you made as a result of using this checklist?

Changes in the Method section.

How much time did you spend on going through the checklist INCLUDING making changes in your manuscript *

As a result of using this checklist, do you think your manuscript has improved?
*

- yes
- no
- Muu:

Would you like to become involved in the CONSORT EHEALTH group?
This would involve for example becoming involved in participating in a workshop and writing an "Explanation and Elaboration" document

- yes
- no
- Muu:

Any other comments or questions on CONSORT EHEALTH

STOP - Save this form as PDF before you click submit

To generate a record that you filled in this form, we recommend to generate a PDF of this page (on a Mac, simply select "print" and then select "print as PDF") before you submit it.

When you submit your (revised) paper to JMIR, please upload the PDF as supplementary file.

Don't worry if some text in the textboxes is cut off, as we still have the complete information in our database. Thank you!

Final step: Click submit !

Click submit so we have your answers in our database!

Lähetä

Palvelun tarjoaa Google-dokumentit

Ilmoita väärinkäytöstä - Palveluehdot - Lisäehdot
